# Natural reversal of pulmonary vascular remodeling and right ventricular remodeling in SU5416/hypoxia-treated Sprague-Dawley rats

**DOI:** 10.1371/journal.pone.0182551

**Published:** 2017-08-15

**Authors:** Makhosazane Zungu-Edmondson, Nataliia V. Shults, Oleksiy Melnyk, Yuichiro J. Suzuki

**Affiliations:** Department of Pharmacology and Physiology, Georgetown University Medical Center, Washington, DC, United States of America; University of Illinois at Chicago College of Medicine, UNITED STATES

## Abstract

**Aims:**

Pulmonary arterial hypertension (PAH) is a lethal disease and improved therapeutic strategies are needed. Increased pulmonary arterial pressure, due to vasoconstriction and vascular remodeling, causes right ventricle (RV) failure and death in patients. The treatment of Sprague-Dawley rats with SU5416 injection and exposure to chronic hypoxia for three weeks followed by maintenance in normoxia promote progressive and severe PAH with pathologic features that resemble human PAH. At 5–17 weeks after the SU5416 injection, PAH is developed with pulmonary vascular remodeling as well as RV hypertrophy and fibrosis. The present study investigated subsequent events that occur in these PAH animals.

**Methods & results:**

At 35 weeks after the SU5416 injection, rats still maintained high RV pressure, but pulmonary vascular remodeling was significantly reduced. Metabolomics analysis revealed that lungs of normal rats and rats from the 35-week time point had different metabolomics profiles. Despite the maintenance of high RV pressure, fibrosis was resolved at 35-weeks. Masson’s trichrome stain and Western blotting monitoring collagen 1 determined 12% fibrosis in the RV at 17-weeks, and this was decreased to 5% at 35-weeks. The level of myofibroblasts was elevated at 17-weeks and normalized at 35-weeks.

**Conclusions:**

These results suggest that biological systems possess natural ways to resolve pulmonary and RV remodeling. The resolution of RV fibrosis appears to involve the reduction of myofibroblast-dependent collagen synthesis. Understanding these endogenous mechanisms should help improve therapeutic strategies to treat PAH and RV failure.

## Introduction

Pulmonary arterial hypertension (PAH) remains a fatal disease without a cure [1,2]. In PAH, pulmonary vascular resistance is increased, exerting strain on the right ventricle (RV), which leads to right heart failure and death [3–5]. If untreated, the median survival of PAH patients has been reported to be 2.8 years from the time of diagnosis (3-year survival: 48%) [1,6]. Even with currently available drugs, the 3-year survival of PAH has been reported to be only 58–75% [7–9]. The initial response to chronic pressure overload is to thicken the RV wall to increase the force of cardiac muscle contraction. However, this compensatory RV hypertrophic event ultimately transitions to RV failure, which is the major cause of death among PAH patients [10]. By the time patients are diagnosed with PAH, their RVs are usually already affected. However, currently available drugs are all vasodilators. Therapeutic strategies that directly repair the affected RV will have a significant impact on health and reduce the morbidity and mortality associated with PAH.

Despite RV failure being the major cause of death among PAH patients, RV pathophysiology is not well understood [11]. We should consider that the mechanisms of right-sided and left-sided heart failure processes could be quite different. The concentric hypertrophy of the LV usually transitions to eccentric hypertrophy, leading to the thinning of the LV wall. By contrast, concentric hypertrophy is the hallmark of the failed RV in *cor pulmonale* [12,13]. It is not clear whether the RV benefits from therapies that are designed to treat LV failure [11]. Since the cardiomyocytes of the LV and RV are developed from different progenitor cells [14,15] and RVs pump the blood against a wide range of pressure (~100 mmHg in utero and ~10 mmHg after birth), the biology of the RV is expected to be different from that of the LV.

The treatment of rats with SU5416, an inhibitor of vascular endothelial growth factor (VEGF) receptor, and chronic hypoxia develops severe PAH [16,17]. Vascular pathology in these rats closely mimics that observed in human PAH patients. These animals also exhibit signs of RV failure, including decreased RV capillary density and induced myocardial apoptosis and fibrosis [18]. In this model, rats are injected with SU5416, subjected to hypoxia for 3 weeks and then maintained in normoxia for various durations. Most published studies have examined animals at 5–8 weeks after the SU5416 injection [16,18–24]. Abe et al [17] studied pulmonary vessels at 13–14 weeks after the SU5416 administration, which is the longest duration after the SU5416 injection studied. Information about the RV is only available at 5 or 6 weeks after the SU5416 injection [18–21]. Thus, the present study examined SU5416/hypoxia-treated rats at 5, 8, 17 and 35 weeks after the SU5416 injection with 3 weeks of hypoxia.

## Materials and methods

### Animal treatment

Male Sprague-Dawley rats (Charles River Laboratories International, Inc., Wilmington, MA, USA) were subcutaneously injected with SU5416 (20 mg/kg body weight; TOCRIS, Minneapolis, MN, USA), maintained in hypoxia for three weeks [20,21] and then in normoxia for 2, 5, 14 or 32 weeks. Animals were subjected to hypoxia in a chamber (30”w x 20”d x 20”h) regulated by an OxyCycler Oxygen Profile Controller (Model A84XOV; BioSpherix, Redfield, NY, USA) set to maintain 10% O_2_ with an influx of N_2_ gas, located in the animal care facility at the Georgetown University Medical Center [21,25]. Ventilation to the outside of the chamber was adjusted to remove CO_2_, such that its level did not exceed 5,000 ppm. Control animals were subjected to ambient 21% O_2_ (normoxia) in another chamber. Animals were fed normal rat chow.

At the end of the experiments, some rats were anesthetized with the intraperitoneal injection of xylazine (10 mg/kg body weight) and ketamine (100 mg/kg body weight). They were then intubated and mechanically ventilated with a volume-controlled Inspira Advanced Safety Ventilator (Harvard Apparatus, Holliston, MA, USA). Rats were maintained on a heat pad and the temperature was kept at 37°C by using a TR-200 Temperature Controller connected to a rectal probe (Fine Scientific Tools, North Vancouver, Canada). After a thoracotomy, a Millar catheter (1.4 F) was inserted into the RV apex. RV pressure signals were recorded by using PowerLab with Chart 5 software (ADInstruments, Colorado Springs, CO, USA). Animals were then euthanized by excising the heart and lung.

The Georgetown University Animal Care and Use Committee approved all animal experiments, and the investigation conformed to the National Institutes of Health (NIH) Guide for the Care and Use of Laboratory Animals. A total of 64 rats were used in this study.

### Histological measurements

Heart and lung tissues were immersed in buffered 10% formalin at room temperature, and were embedded in paraffin. Paraffin-embedded tissues were cut and mounted on glass slides. Tissue sections were subjected to hematoxylin and eosin (H&E) stain, Verhoeff-Van Gieson stain (VVG), Masson’s trichrome stain, and immunohistochemistry using α-smooth muscle actin (Abcam, Cambridge, UK) and periostin (MyBioSource, San Diego, CA).

### Metabolomics

Lung tissues were homogenized in 50% methanol containing internal standards and then centrifuged at 13,000 rpm for 10 min. An equal volume of chilled acetonitrile was added to each sample tube, and then was vortexed and incubated overnight at -20°C. Next, the tubes were again centrifuged at 13,000 rpm for 10 min at room temperature and the supernatant was transferred to fresh tubes and dried under vacuum. The dried metabolite mixture was resuspended in 100 μl of 50% methanol for mass spectrometry analysis.

Samples were injected onto a reverse-phase column using an Acquity ultra-performance liquid chromatography (UPLC) system (Waters Corporation, USA). Mass spectrometry was performed by using a Quadrupole-time-of-flight mass spectrometer operating in either negative or positive electrospray ionization mode with a capillary voltage of 3.2 kV and a sampling cone voltage of 35 V. Data were acquired in centroid mode with a mass range from 50 to 850 m/z for TOF-MS scanning.

### Western blotting

RVs were homogenized and protein gel electrophoresis samples were prepared as previously described [21,25]. Equal protein amounts of samples were electrophoresed through a reducing SDS polyacrylamide gel and electroblotted onto a nitrocellulose membrane. Membranes were blocked and incubated with antibodies for cleaved caspase-3 (Cell Signaling Technology, Inc., Danvers, MA, USA), glutaraldehyde-3-phosphate-dehydrogenase (G3PDH), and collagen A1 (COLA1) (Santa Cruz Biotechnology, Inc., Dallas, TX, USA) and levels of proteins were detected by using horseradish peroxidase-linked secondary antibodies and an Enhanced Chemiluminescence System (GE Healthcare Bio-Sciences, Pittsburgh, PA, USA).

### Statistical analysis

Means and standard errors were calculated. Comparisons between two groups were analyzed by using a two-tailed Student’s *t* test and comparisons between three or more groups were analyzed by using one-way analysis of variance (ANOVA) with a Student-Newman-Keuls post-hoc test using the GraphPad Prism (GraphPad Software, Inc., La Jolla, CA, USA), in accordance with the Kolmogorov-Smirnov test for normality. *P* < 0.05 was considered to be significant.

## Results

**Fig 1:**
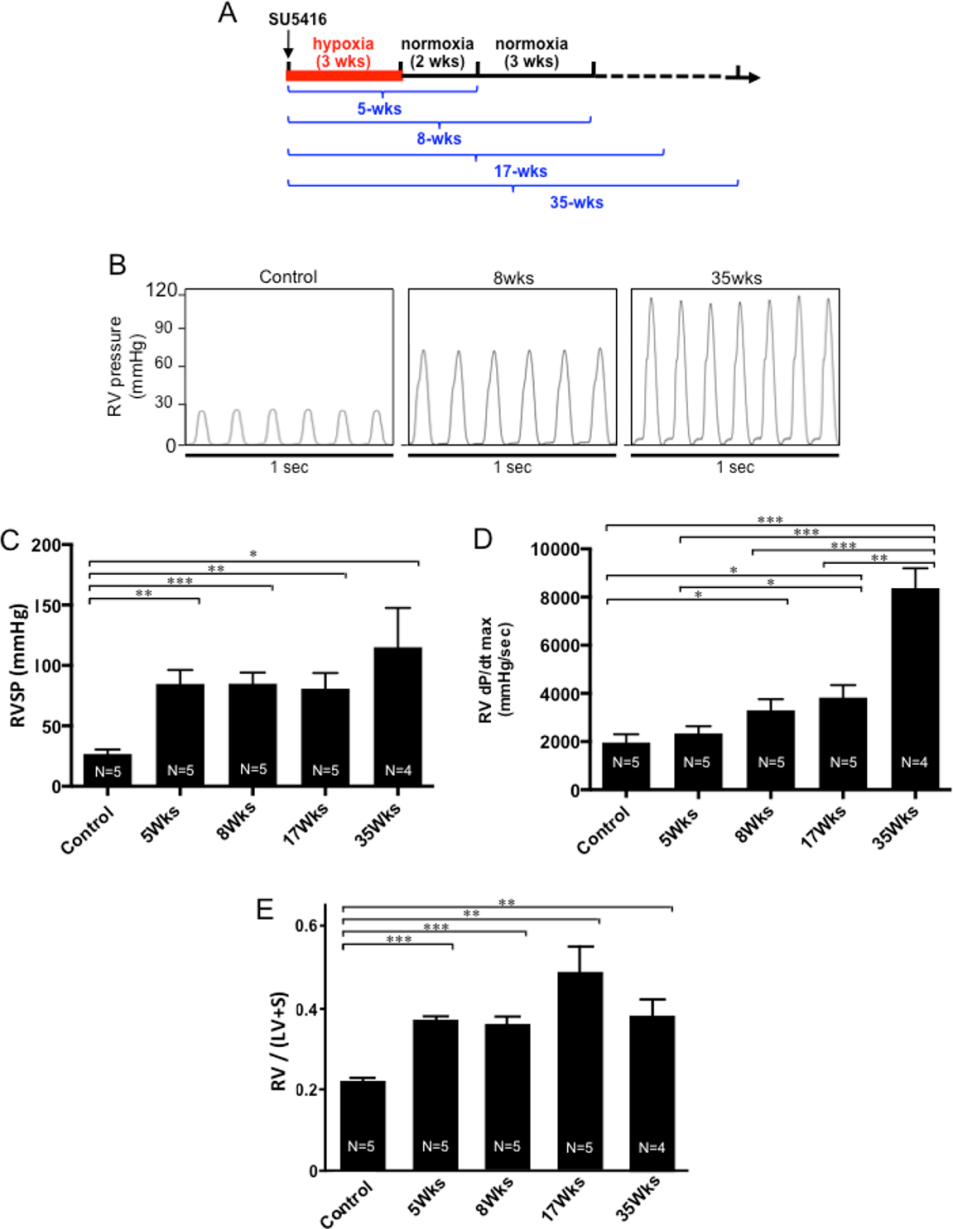
RV hemodynamics. SU5416-injected rats were subjected to 3 weeks in hypoxia and then maintained in normoxia for 2, 5, 14 or 32 weeks (5-, 8-, 17- and 35-wks time points, respectively). Animals were anaesthetized, ventilated and the chest opened. A Millar catheter was inserted into the RV apex and hemodynamic measurements were performed. (A) Flow diagram of the experiments. (B) Representative hemodynamic traces. (C) Means ± SEM of RVSP. (D) Means ± SEM of dP/dt_max_. (E) Means ± SEM of the ratio of RV weight to LV + septum weight. *Significantly different from each other at *P* < 0.05. **Significantly different from each other at *P* < 0.01. ***Significantly different from each other at *P* < 0.001.

Rats were injected with SU5416, exposed to hypoxia for 3 weeks and maintained in the normoxic condition for 2, 5, 14 or 32 weeks thereafter. We designated these rats to be 5-, 8-, 17- and 35-week time points, respectively (Fig 1A). Initially, there were eight rats in each group. In all SU5416/hypoxia groups, one rat died at 2 or 3 weeks after the SU5416 administration. In all of the 8-, 17- and 35-week groups, one rat died at 4 or 5 weeks. For the 17- and 35-week groups, two rats and one rat, respectively, died between 10 and 12 weeks. Finally, for the 35-week group, one rat died at 32 weeks after the SU5416 injection. Thus, the incidence of death was consistent in all groups and occurred throughout the study. No control rats without PAH died up to 35 weeks.

Representative hemodynamic traces are shown in Fig 1B. RV systolic pressure (RVSP) increased to ~80 mmHg by 5 weeks after the SU5416 injection, and this level of pressure was maintained up to 17 weeks. At 35 weeks, mean RVSP was over 100 mmHg (Fig 1C). The RV contractility as indicated by dP/dt_max_ was dramatically increased at 35 weeks (Fig 1D). Consistent with data of RV pressure, RV hypertrophy as measured by assessing the ratio of the RV weight to LV + septum weight (Fulton Index) was significantly increased by 5 weeks after the SU5416 injection and was sustained thereafter (Fig 1E).

### Pulmonary artery and lung remodeling

**Fig 2:**
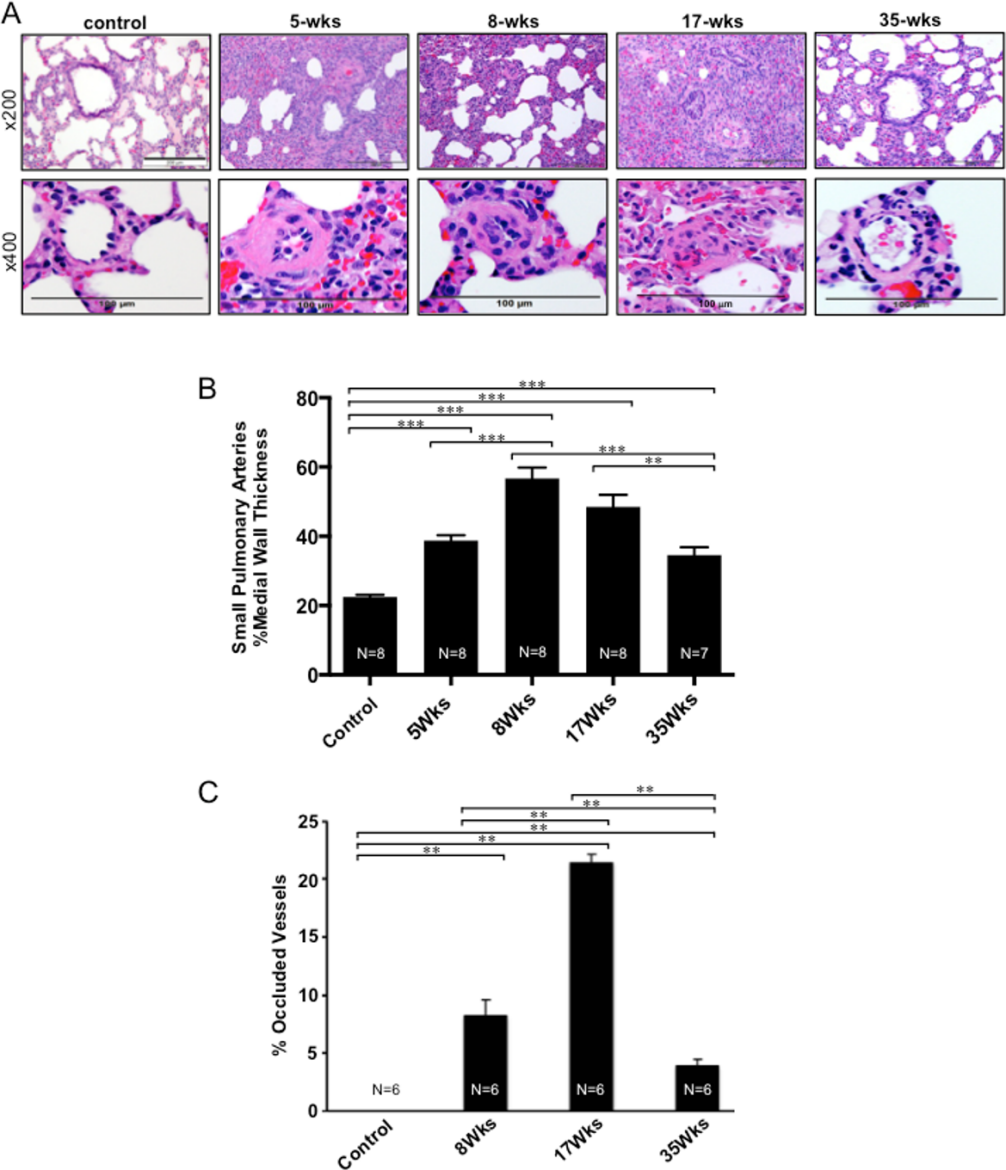
Pulmonary vascular remodeling. SU5416-injected rats were subjected to 3 weeks in hypoxia and then maintained in normoxia for 2, 5, 14 or 32 weeks (5-, 8-, 17- and 35-wks time points, respectively). (A) Representative lung H&E stain results at x200 and x400 magnifications. (B) Means ± SEM of % pulmonary arterial wall thickness. (C) Means ± SEM of % of completely occluded pulmonary arterial vessels (30–100 μm diameter). **Significantly different from each other at *P* < 0.01. ***Significantly different from each other at *P* < 0.001.

The H&E staining results (Fig 2A) and their quantifications demonstrated that the wall thickness (Fig 2B) and the occlusion (Fig 2C) of the small pulmonary artery and arterioles were significantly increased at 5 weeks and progressively increased thereafter. Pulmonary vascular remodeling is characterized by the thickening of all three layers of the blood vessel wall (adventitia, media, and intima) at 5, 8, and 17 weeks. The lumens of vessels with diameter >50 μm were found to be significantly decreased and those of vessels with diameter <50 μm were completely obliterated. Surprisingly, at 35 weeks, the vessel wall thickness was reduced and the lumens of the pulmonary artery were significantly greater.

**Fig 3:**
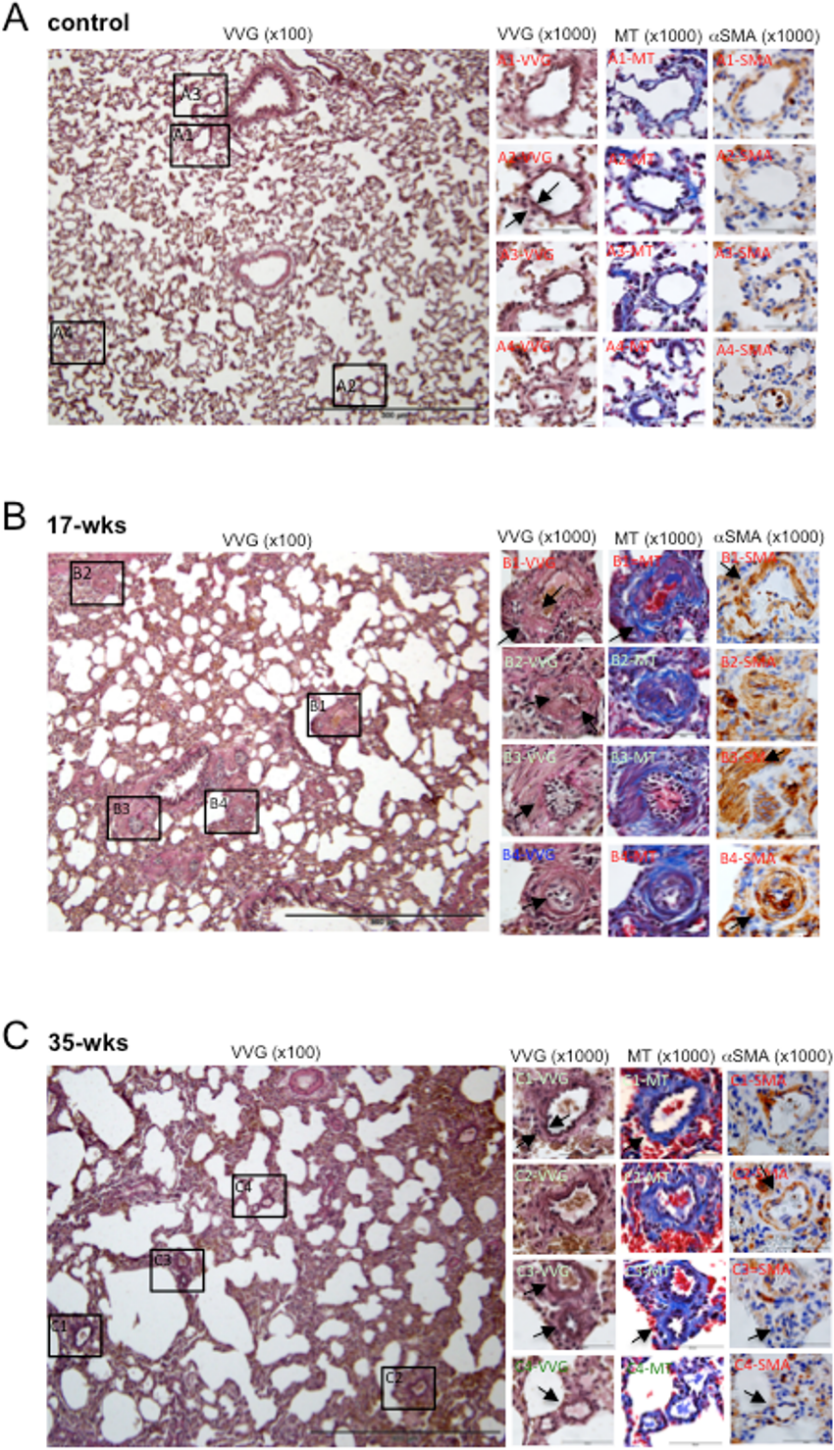
Lung structure. SU5416-injected rats were subjected to 3 weeks in hypoxia and then maintained in normoxia for 14 or 32 weeks (17- and 35-wks time points, respectively). The large panels on the left are representative VVG stains at x100 magnification. Sections of different sizes of pulmonary arteries (50-150nm) are enlarged (x1000) on the right, showing VVG stain, Masson’s trichrome (MT) stain and immunohistochemistry of α-smooth muscle actin (αSMA).

As shown in Fig 3A, in the lungs of healthy control rats, the VVG stain revealed well-defined vessel walls (arrows) with internal elastic lamina (brown). Masson's trichrome stain showed connective tissue and normal collagen deposition (blue). Immunohistochemistry demonstrated α-smooth muscle actin (brown) in the smooth muscle layer in the walls of vessels.

By contrast, thickened pulmonary vessels in PAH animals were accompanied by overall changes in the lung structure including the loss of alveolar space, especially at 8 weeks. The lung sections of rats with PAH at the 17-week time point (Fig 3B) exhibited atelectasis and the emphysematous expansion of the alveoli. Alveolar septa were thickened with inflammatory infiltrate. The thickening of the vessel walls, reduced lumens, and perivascular inflammatory infiltrate were also observed. The VVG stain showed that the medial wall had thickened by an increased amount of collagen and elastic fibers in vessels with diameters 50–100 μm (arrows in Fig 3B Panel B1-VVG) as well as an increase in the amount of collagen and elastic fibers with the destruction of elastic membranes of small vessels (arrows in Fig 3B Panel B2-VVG). The shirring of the elastic membrane had disappeared (Fig 3B Panel B2-VVG), accompanied by the concentric cellular laminar neointimal proliferation, complete occlusion of small pulmonary arteries (arrow in Fig 3B Panel B3-VVG) and nearly complete occlusion of small pulmonary arteries (arrow in Fig 3B Panel B4-VVG) by concentric endothelial cell proliferation.

Masson’s trichrome stain demonstrated perivascular fibrosis (arrow in Panel B1-MT) and increased collagen fibers in the adventitial layer (Panel B2-MT). Immunohistochemistry revealed the hypertrophy and hyperplasia of smooth muscle with an increase in α-smooth muscle actin expression (arrows in Panels B1-SMA and B3-SMA) and small arteries with a thick muscularized intima (muscularization of small pulmonary vessels) (arrow in Panel B4-SMA).

These pathologic features were found to be normalized at 35 weeks. Fig 3C shows that the atelectasis and emphysematous expansion of alveoli was less pronounced compered with the 17-week time point. The lumens of large and small pulmonary arteries were increased. The examination of the high magnification of the VVG stain demonstrated the reduced thickness of media vessels (Fig 3C Panel C1-VVG); the reduction in the thickness of the media was largely due to the decreased amounts of elastic and collagen fibers (arrow in Fig 3C Panel C1-VVG; also see Fig 3C Panel C1-MT of Masson’s trichrome stain), thin intima of small pulmonary arteries and shirring of elastic membranes (Fig 3C Panels C3-VVG and C4-VVG). Masson’s trichrome stain indicated that, at 35 weeks, perivascular fibrotization was still observed; however, it was less pronounced compered with the 17-week time point (Fig 3C Panels C3-MT and C4-MT). The hypertrophy and hyperplasia of smooth muscle and the expression of α-smooth muscle actin in small pulmonary vessels were found to be less or disappeared in the lungs of 35-week rats compared to 17-weeks (arrows), suggesting the remuscularization of small pulmonary vessels (Fig 3C Panels C3-SMA and C4-SMA).

**Fig 4:**
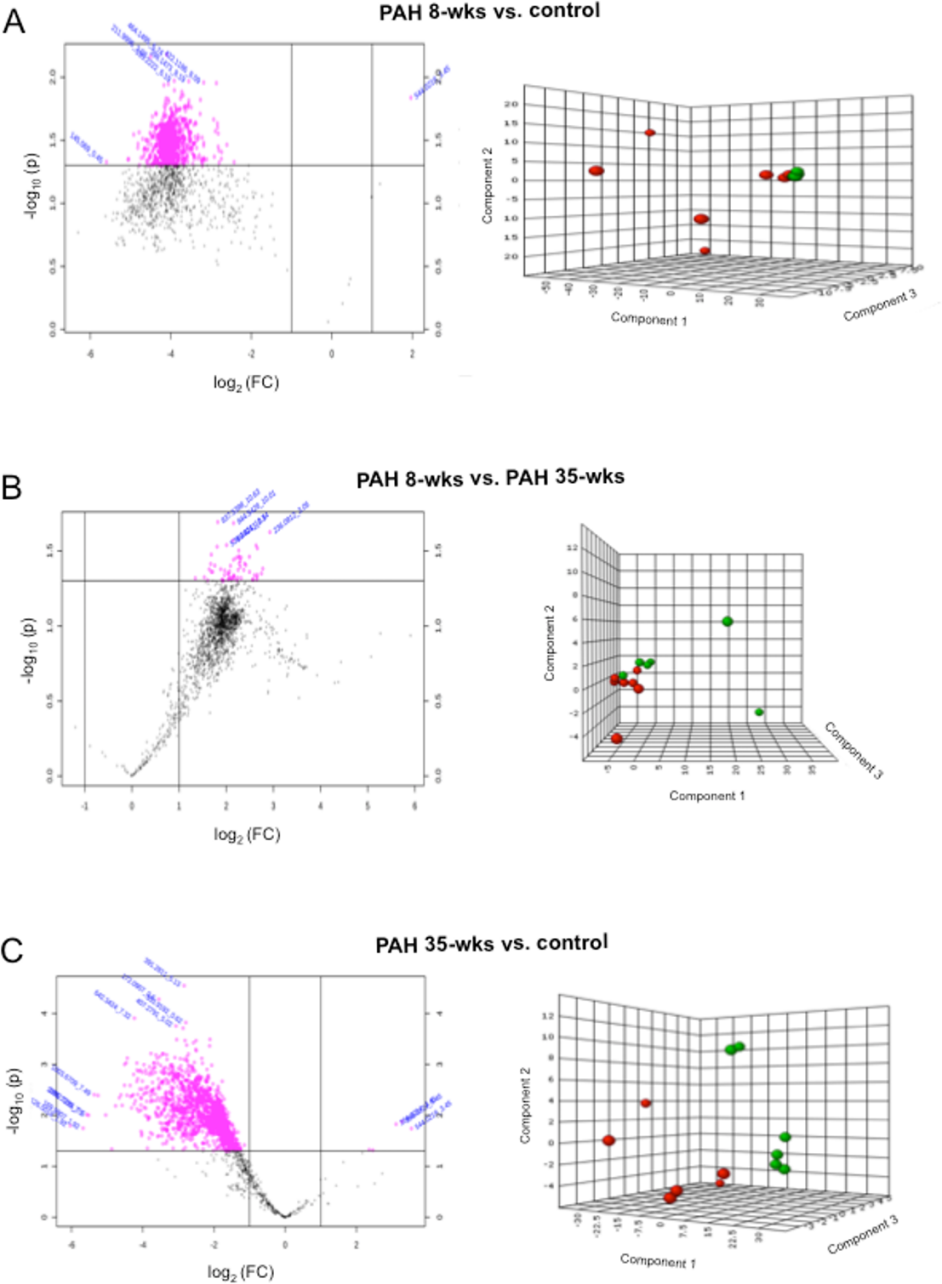
Metabolomics analysis of the lung. SU5416-injected rats were subjected to 3 weeks in hypoxia and then maintained in normoxia for 5 weeks (8-wks time point) or 32 weeks (35-wks time point). Metabolomics analysis was performed in the lung tissues. Volcano plots (on the left) and Partial least squares Discriminant Analysis (PLS-DA; on the right) for (A) 8-weeks PAH (green circles) vs. normal control (red circles), (B) 8-weeks PAH (green circles) vs. 35-weeks PAH (red circles) and (C) 35-weeks PAH (green circles) vs. normal control (red circles) are shown. Dot volcano plots represent fold changes (FC) on the x-axis and the statistical significance in *P* value on the y-axis. Pink dots represent values with *P*≤0.05 and changes of greater than twofold.

We asked whether this apparent reversal of pulmonary vascular remodeling represents the normalization of the remodeled lung or conversion to a different entity. Global metabolites were analyzed in the lungs of control rats as well as PAH rats at the 8- and 35-week time points using mass spectrometry. Statistically significant differences at *P* < 0.05 and ≥ 2-fold changes were analyzed by using metabolomics analysis. The PLS-DA plots show that metabolites were found to be different between PAH lungs at the 8-week time point and healthy controls (Fig 4A), between PAH lungs at 8 and 35 weeks (Fig 4B) and between PAH at 35-week time point and controls (Fig 4C). Metabolites that were significantly increased from 8-week PAH with remodeled lungs to 35-week PAH with reduced remodeling included glyceraldehyde/lactic acid (4.4-fold increase in 35-week PAH from 8 weeks); glucose/fructose (4.3-fold increase), tryptophan (4.2-fold increase), arginine (5.3-fold increase), phenylalanine (4.5-fold increase), leucyl-proline/isoleucyl proline (6.7-fold increase) and heme (5.9-fold increase).

The metabolomics analysis showed that 35-week PAH and control lungs were clearly different, indicating that the event is not a reversal process to the original entity but rather the conversion of the remodeled lung to another entity with reduced remodeling. Further, 35-week PAH lungs had 5.1-fold higher nicotinic acid, 3-fold lower retinoic acid, 2-fold lower cystine, 2-fold lower ascorbyl palmitate, 5.6-fold lower uric acid and 10-fold lower maleic acid/fumaric acid.

Complete metabolomics data are found in S1 Supporting Information "Metabolomics Database Search 1", S2 Supporting Information "Metabolomics Database Search 2", S3 Supporting Information "Metabolomics Database Search 3", S4 Supporting Information "Metabolomics Database Search 4", S5 Supporting Information "Metabolomics Dataset 1", S6 Supporting Information "Metabolomics Dataset 2", and S7 Supporting Information "Metabolomics MS results").

### RV remodeling

**Fig 5:**
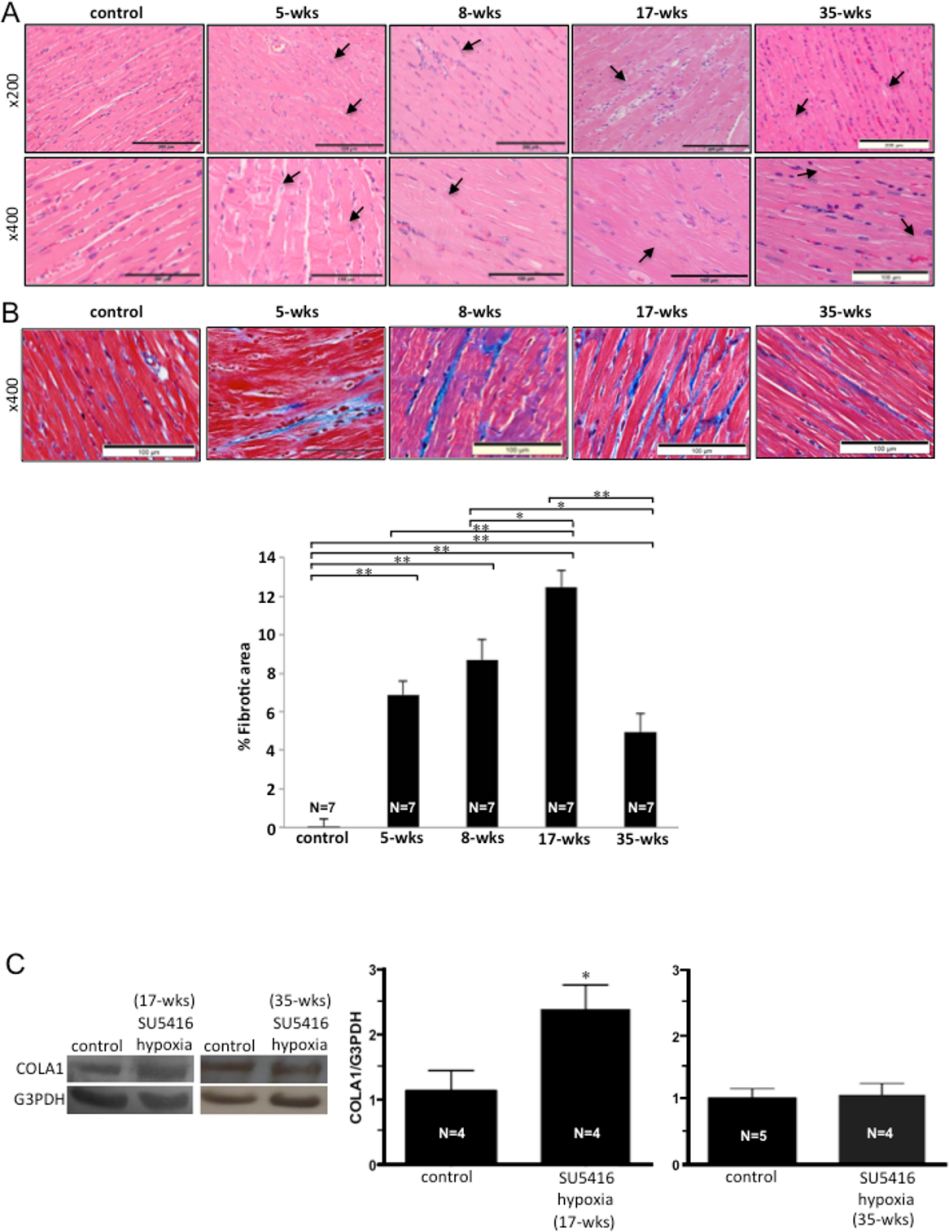
RV damage and fibrosis. SU5416-injected rats were subjected to 3 weeks in hypoxia and then maintained in normoxia for 2, 5, 14 or 32 weeks (5-, 8-, 17- and 35-wks time points, respectively). (A) Representative H&E staining results of the RV tissue sections. (B) Masson’s trichrome stain results in the RVs. Blue stains indicate fibrotic areas. Bar graph represents means ± SEM of % fibrotic area (N = 7). *Significantly different from each other at *P* < 0.05. **Significantly different from each other at *P* < 0.01. (C) Western blotting showing COLA1 expression as an indicator of fibrosis in the RVs at 17 and 35 weeks after the SU5416 injection. Bar graph represents means ± SEM of the ratio of COLA1 to G3PDH. *Significantly different from the control value.

The analysis of H&E-stained hearts of the control rats (Fig 5A) showed the syncytium of myocardial fibers with central nuclei and faint pink intercalated discs across some of the fibers. Red blood cells were in the capillaries between the fibers. By contrast, the hearts of rats with PAH displayed the myofiber disarray, degeneration of myocytes, polymorphic cardiomyocytes, hypertrophied cardiomyocytes, hypereosinophilia and hyperbasophilia of cardiomyocytes (arrows in 5 weeks), focal lesions of cardiomyocytes (arrows in 8 and 17 weeks, magnification x200), contractures of cardiomyocytes (arrows in 5 weeks), myocytolysis (arrows in 8 weeks), loss of myofibers striations, wavy arrangement of myofiber (arrows in 17 weeks, magnification x400), edema of cytoplasm, and indistinct nucleus borders in cardiomyocytes. In the RVs of rats at the 35-week time point, normal myofibers with clear striation were observed. However, small microfocal lesions (arrows in 35 weeks, magnification x200) and alterations of cardiomyocytes in the form of granular dystrophy (arrows in 35 weeks, magnification x400) were observed.

Cardiac fibrosis was promoted by SU5416/hypoxia treatment in the RVs at 5 weeks (to 7% fibrotic area), persisted at 8 weeks and further increased at 17 weeks to 12% fibrotic area (Fig 5B) as visualized in Masson’s trichrome-stained heart sections. In 35-week RVs, however, the extent of RV fibrosis decreased to 5%. Western blotting using collagen 1A as a marker of fibrosis also suggested that while 17-week RVs had significantly higher collagen 1A expression than the controls, that of 35-week RVs was comparable to the controls (Fig 5C).

**Fig 6:**
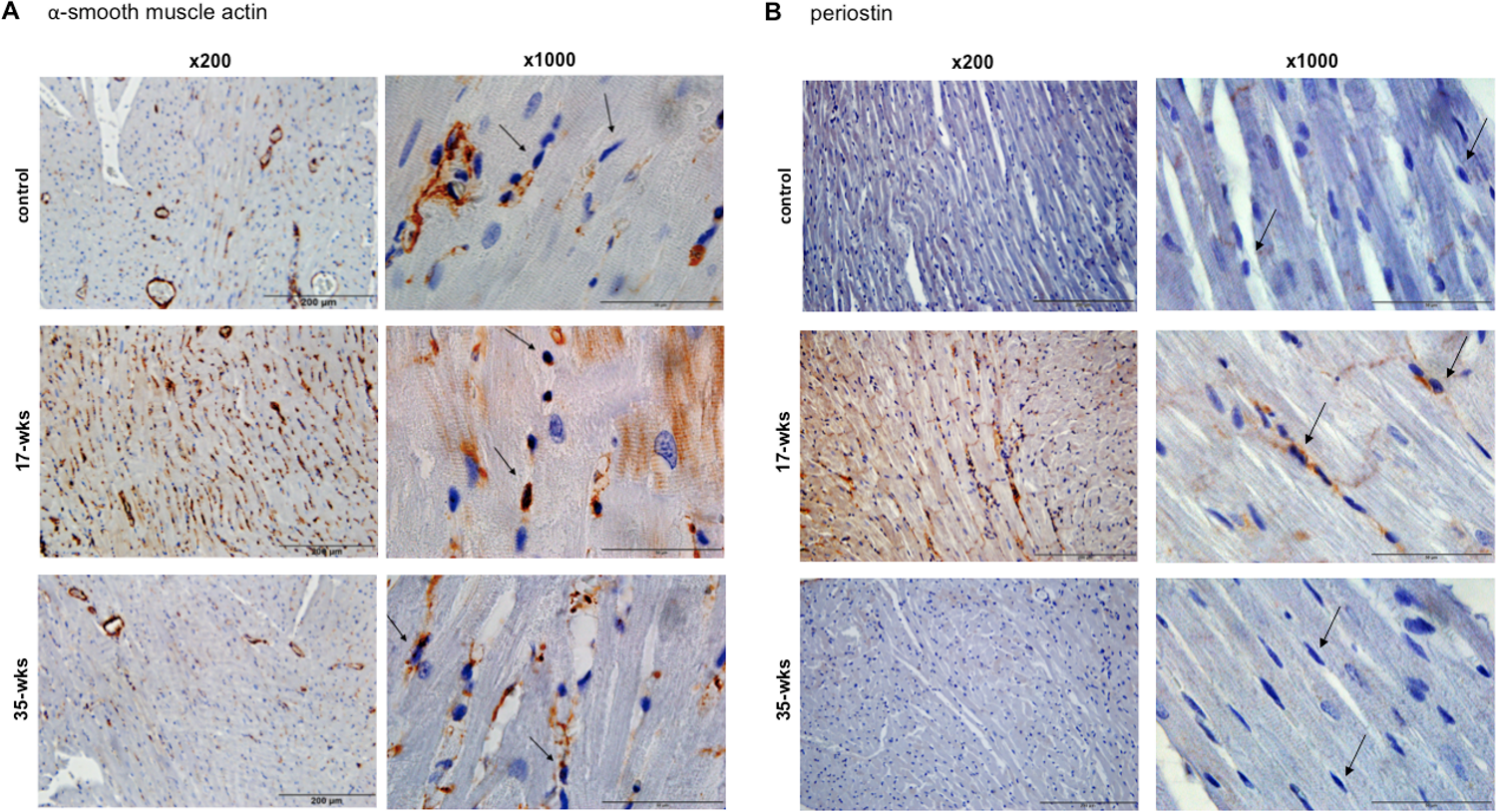
Myofibroblasts in the RV. SU5416-injected rats were subjected to 3 weeks in hypoxia and then maintained in normoxia for 14 or 32 weeks (17- and 35-wks time points, respectively). Hearts were fixed in formalin and embedded in paraffin. Longitudinal sections of the RV myocardium were subjected to immunohistochemistry using the antibody against (A) α-smooth muscle actin and (B) periostin. Results are presented at x200 and x1,000 magnifications. Scale bars indicate 200 μm for x200 and 50 μm for x1,000. The brown stains indicate the expression of α-smooth muscle actin or periostin. Arrows depict that neither α-smooth muscle actin nor periostin stains are present in fibroblasts of the normal RV; brown α-smooth muscle actin and periostin stains are present overlapping nuclear stains of fibroblasts at 17-wks; and α-smooth muscle actin and periostin stains in fibroblasts are absent at 35-wks.

The reduction of collagen expression at 35-week time point compared to 17-weeks could be due to decreased synthesis or increased degradation. Fig 6 shows that the expression level of α-smooth muscle actin and periostin, markers of myofibroblasts with a high capacity to synthesize collagen, was found to be increased at 17-week but was normalized at 35 weeks. These results suggest that decreased myofibroblasts may be responsible for the resolution of RV fibrosis.

## Discussion

The major findings of the present study are that pulmonary vascular remodeling and RV remodeling can naturally be resolved in rats treated with SU5416 plus hypoxia. These findings have great translational importance because if the endogenous mechanisms of the repair/reversal of pulmonary arteries and RV remodeling are understood, therapeutic agents that mimic such natural pathways may be developed to help PAH patients. In this regard, the SU5416/hypoxia model in Sprague-Dawley rats could serve as a useful system to investigate these mechanisms.

While pinpointing the exact mechanisms through which remodeled pulmonary arteries and RV are repaired will need years of research, the present study suggested that reduced pulmonary remodeling is not due to the reversal to the original normal lung, but it seems that the lung has become a new entity with a distinct metabolomics profile. In this particular case, lung and pulmonary vascular structures appear normalized; however, RV pressure was found to be still elevated, suggesting that pulmonary vasoconstriction may be enhanced.

We further found that while cardiac fibrosis progressively increases from the 5- to 17-week time points in the RVs, these lesions are resolved at 35 weeks even though significantly increased RV pressure is still maintained. Thus, the reversal of RV fibrosis is not because the blood pressure decreased. This is a remarkable naturally-occurring process as we assessed that the degree of RV fibrosis that occurs in this model is as high as 12% of RV mass, indicating that the RV had lost >10% of the functional myocardium. However, the RV contractility, as measured by dP/dt, was not weakened. How fibrotic tissues are eliminated and what replaces fibrosis are key future questions. Since the overall RV mass should be reduced at 35 weeks because of the disappearance of fibrotic regions, it is likely that either hypertrophied already existing RV myocytes, newly generated RV myocytes or both fill the regions that were fibrotic.

The resolution of RV fibrosis, i.e. the reduction of the collagen expression seen at 35-week time point can be due to decreased collagen synthesis or increased collagen degradation. Immunohistochemistry showed that myofibroblasts (as stained with α-smooth muscle actin as well as periostin antibodies) are increased in the highly fibrotic RV compared to normal RV, but myofibroblast levels were normalized in association with the RV fibroblast resolution. Thus, it is likely that the mechanism of the RV fibroblast resolution involves the reduction of collagen-forming myofibroblasts.

This animal model is quite useful for studies of adaptive mechanisms, which occur in response to the remodeling of the lung and heart as discussed in this study. Further, by using this model, we have previously reported our findings that despite dramatic cardiac fibrosis and apoptosis occurring in remodeled RVs, RV contractility is maintained because of the generation of ‘super RV myocytes’ regulated by signaling through calsequestrin 2 [26]. Significantly high RV pressure being maintained at 35 weeks, as shown in the present study, suggests that the reversal/repair of RV remodeling is not merely due to the normalization of pulmonary vascular resistance, making this a unique model for understanding the remarkable mechanism nature offers.

In terms of translational utility, the limitation of this particular model using Sprague-Dawley rats treated with SU5416/hypoxia is that these rats do not die of RV failure; thus, one cannot perform drug studies to investigate the effectiveness on survival. For this purpose, Fischer rats treated with SU5416/hypoxia would be more useful, because these rats die in response to developing PAH [27]. Our examinations of Fischer rats suggest that the cause of death is congestive heart failure (unpublished results). In this regard, Fischer rats do not appear to possess the adaptive mechanisms described here, and the comparison between Sprague-Dawley and Fischer rat strains may be a fruitful approach to define these adaptive mechanisms. Understanding these naturally-occurring adaptive mechanisms should help develop new therapeutic strategies to reverse PAH and RV failure.

## Supporting information

S1 Supporting Information Metabolomics Database Search 1. (XLSX)

S2 Supporting Information Metabolomics Database Search 2. (XLSX)

S3 Supporting Information Metabolomics Database Search 3. (XLSX)

S4 Supporting Information Metabolomics Database Search 4. (XLSX)

S5 Supporting Information Metabolomics Dataset 1. (XLSX)

S6 Supporting Information Metabolomics Dataset 2. (XLSX)

S7 Supporting Information Metabolomics MS results. (PPTX)
