# Supplementary material for: Natural reversal of pulmonary vascular remodeling and right ventricular remodeling in SU5416/hypoxia-treated Sprague-Dawley rats
Source: PLoS One. 2017 Aug 15;12(8):e0182551. doi: 10.1371/journal.pone.0182551 (PMC5557492; doi:10.1371/journal.pone.0182551)
Supplement: S7 Supporting Information — (PPTX) [file pone.0182551.s007.pptx]

## Slide 1
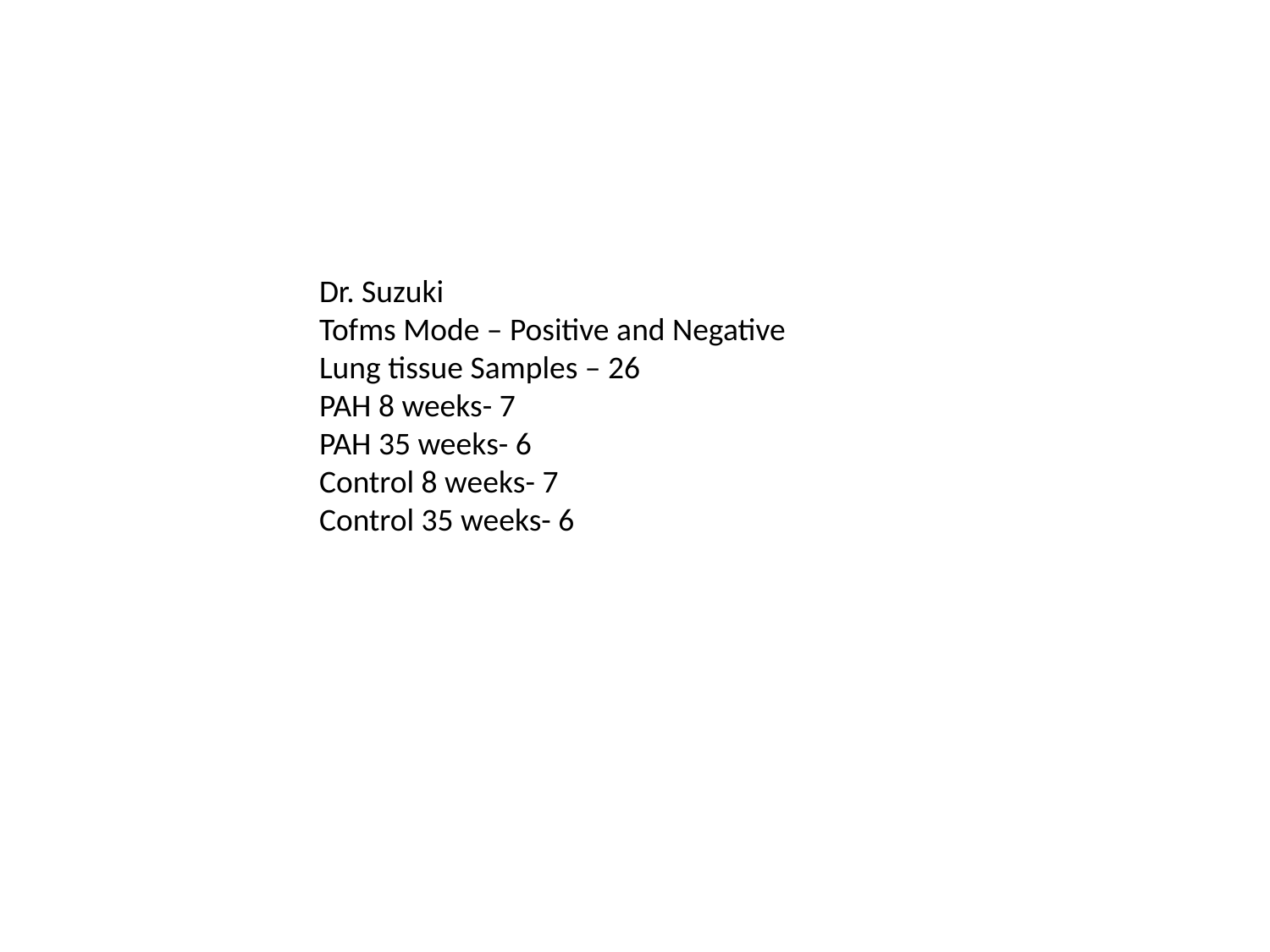

Dr. Suzuki
Tofms Mode – Positive and Negative
Lung tissue Samples – 26
PAH 8 weeks- 7
PAH 35 weeks- 6
Control 8 weeks- 7
Control 35 weeks- 6

## Slide 2
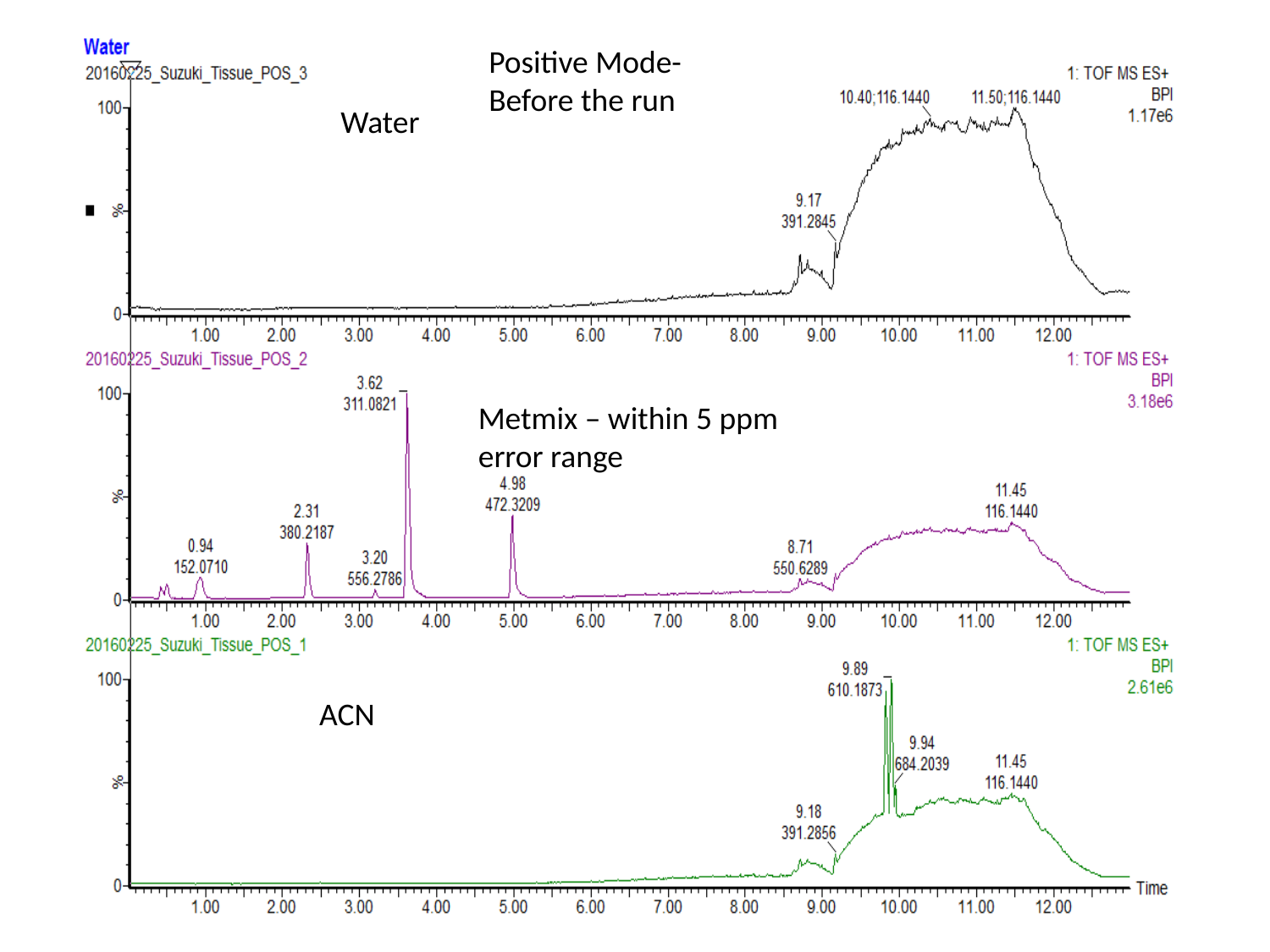

Positive Mode- Before the run
Water
Metmix – within 5 ppm error range
ACN

## Slide 3
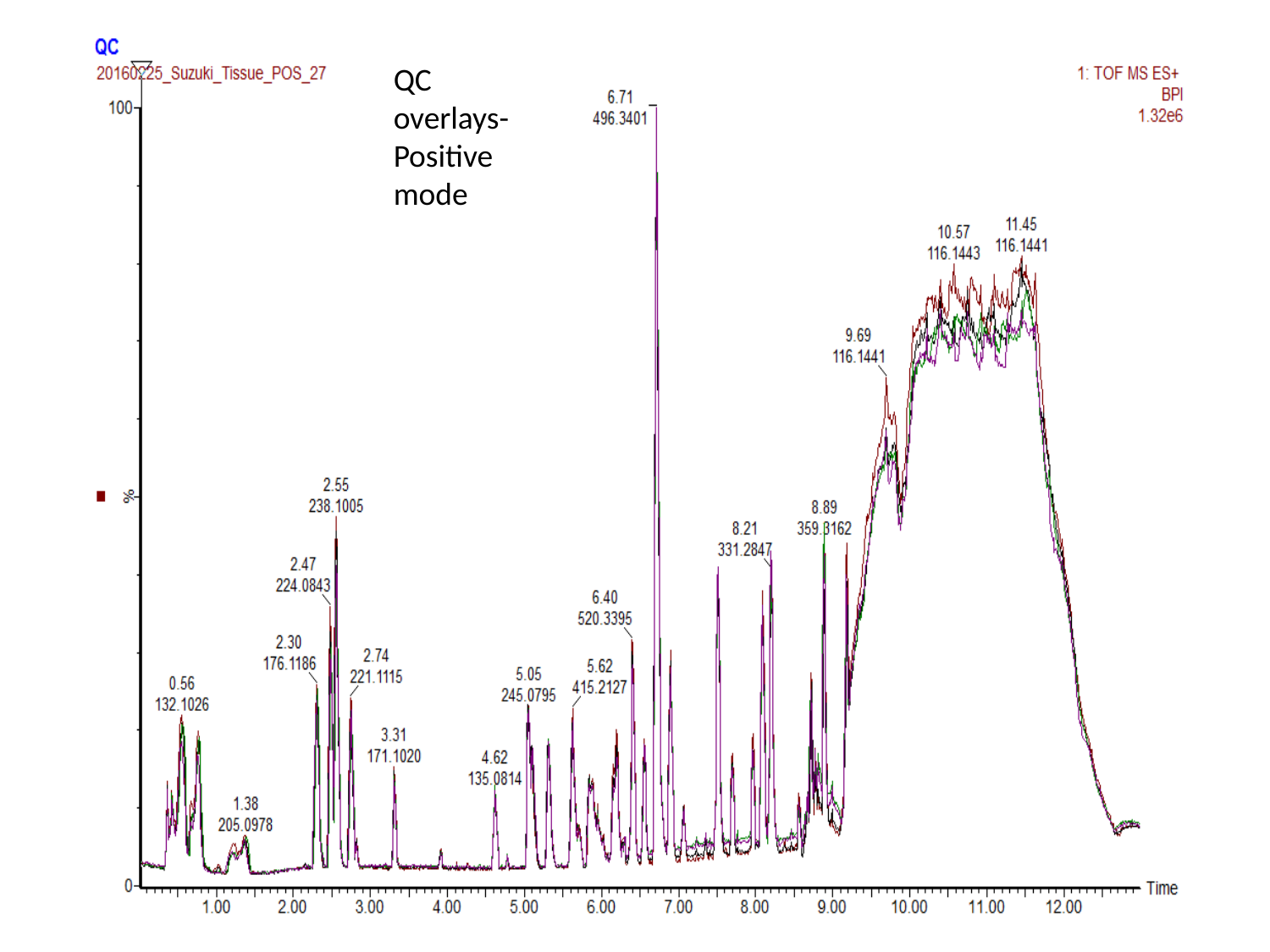

QC overlays- Positive mode

## Slide 4
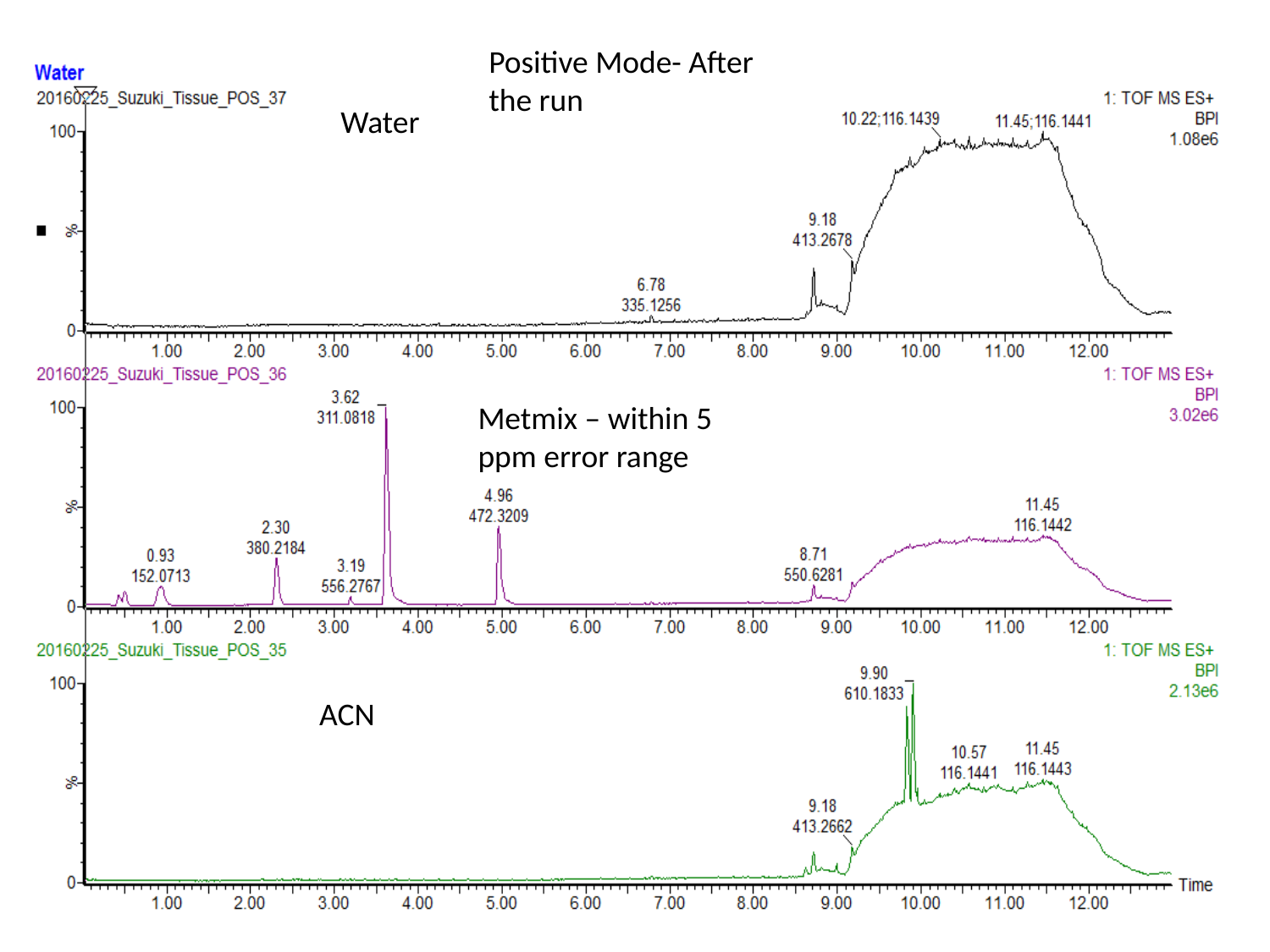

Positive Mode- After the run
Water
Metmix – within 5 ppm error range
ACN

## Slide 5
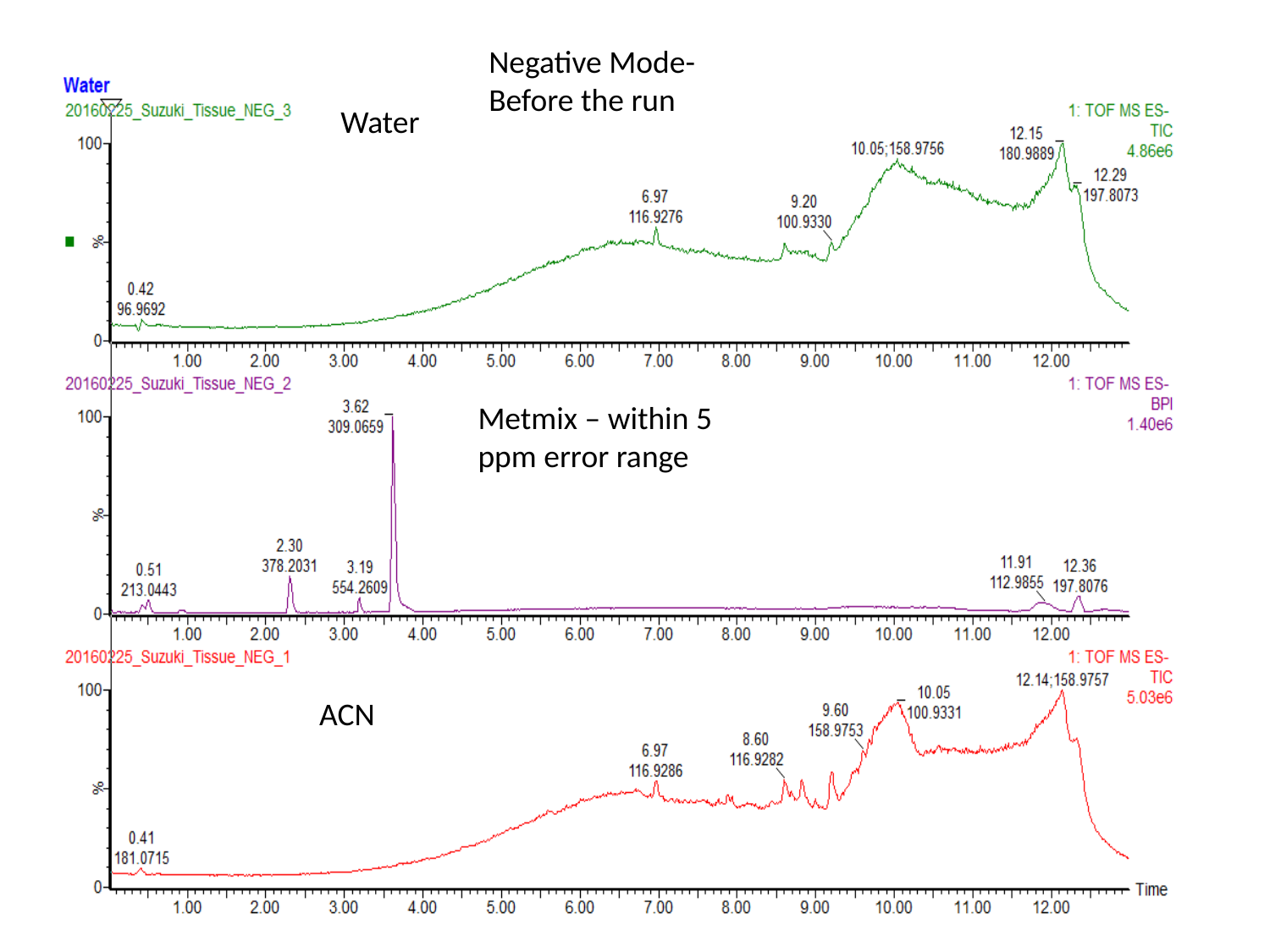

Negative Mode- Before the run
Water
Metmix – within 5 ppm error range
ACN

## Slide 6
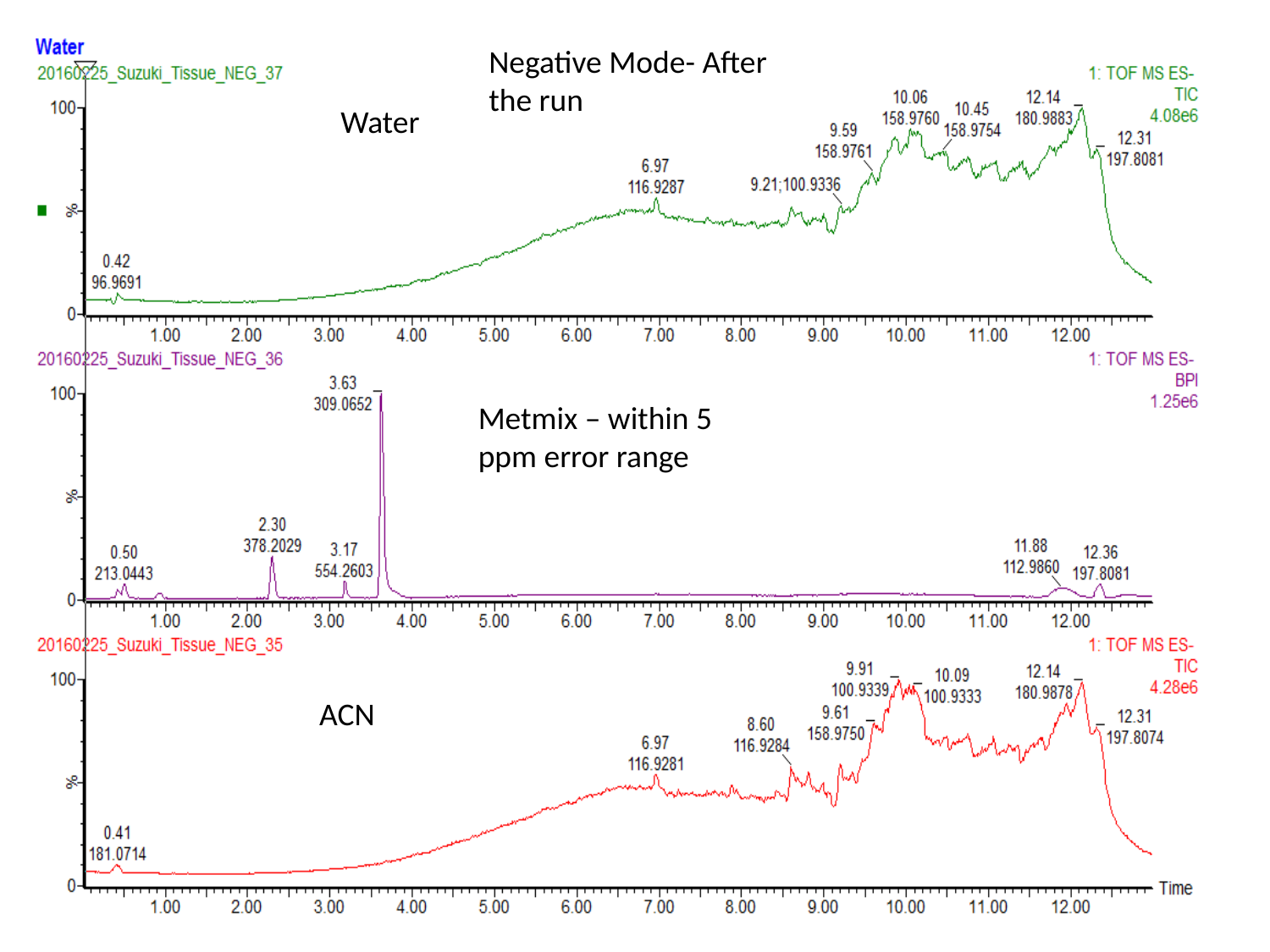

Negative Mode- After the run
Water
Metmix – within 5 ppm error range
ACN

## Slide 7
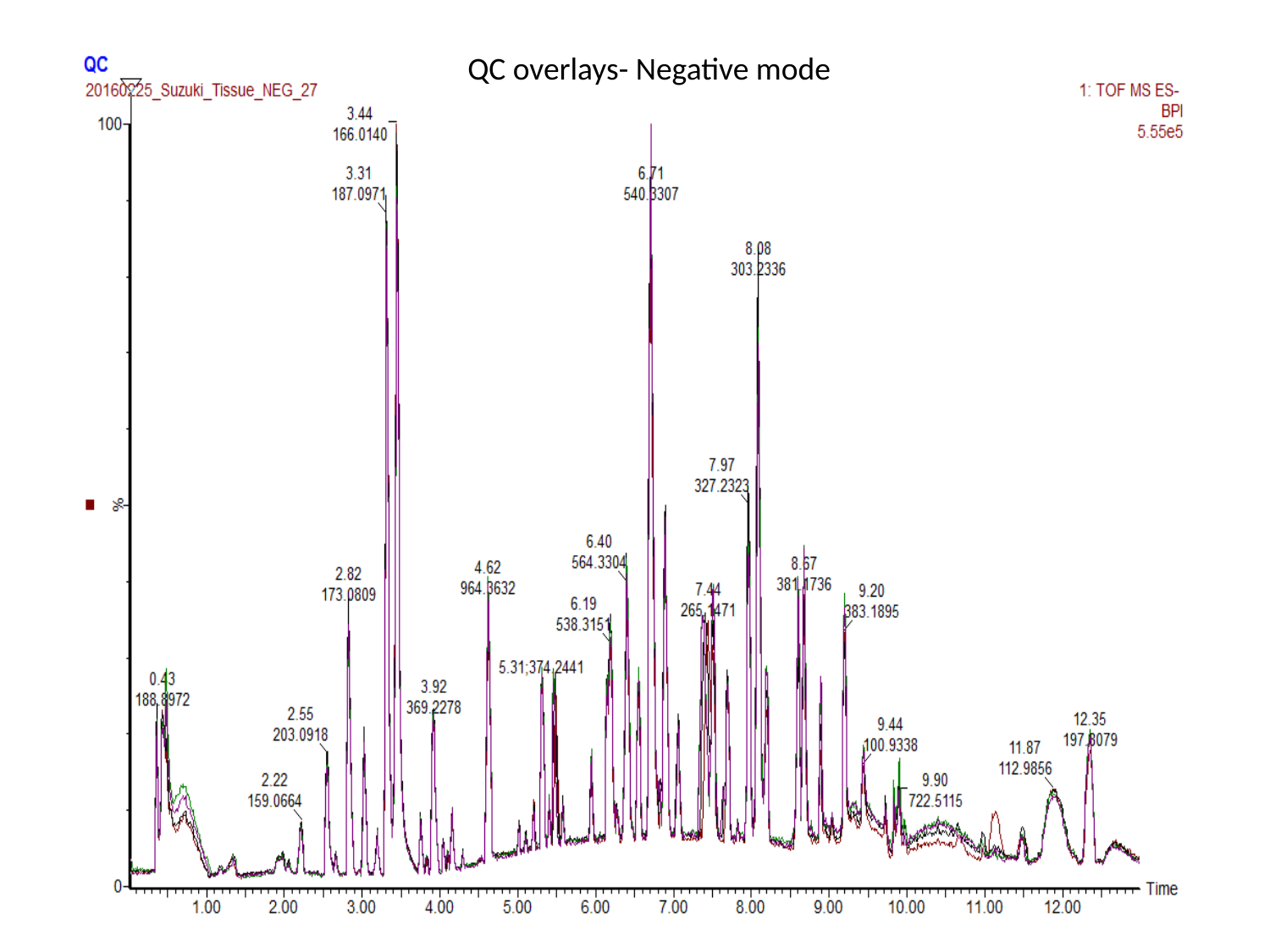

QC overlays- Negative mode

## Slide 8
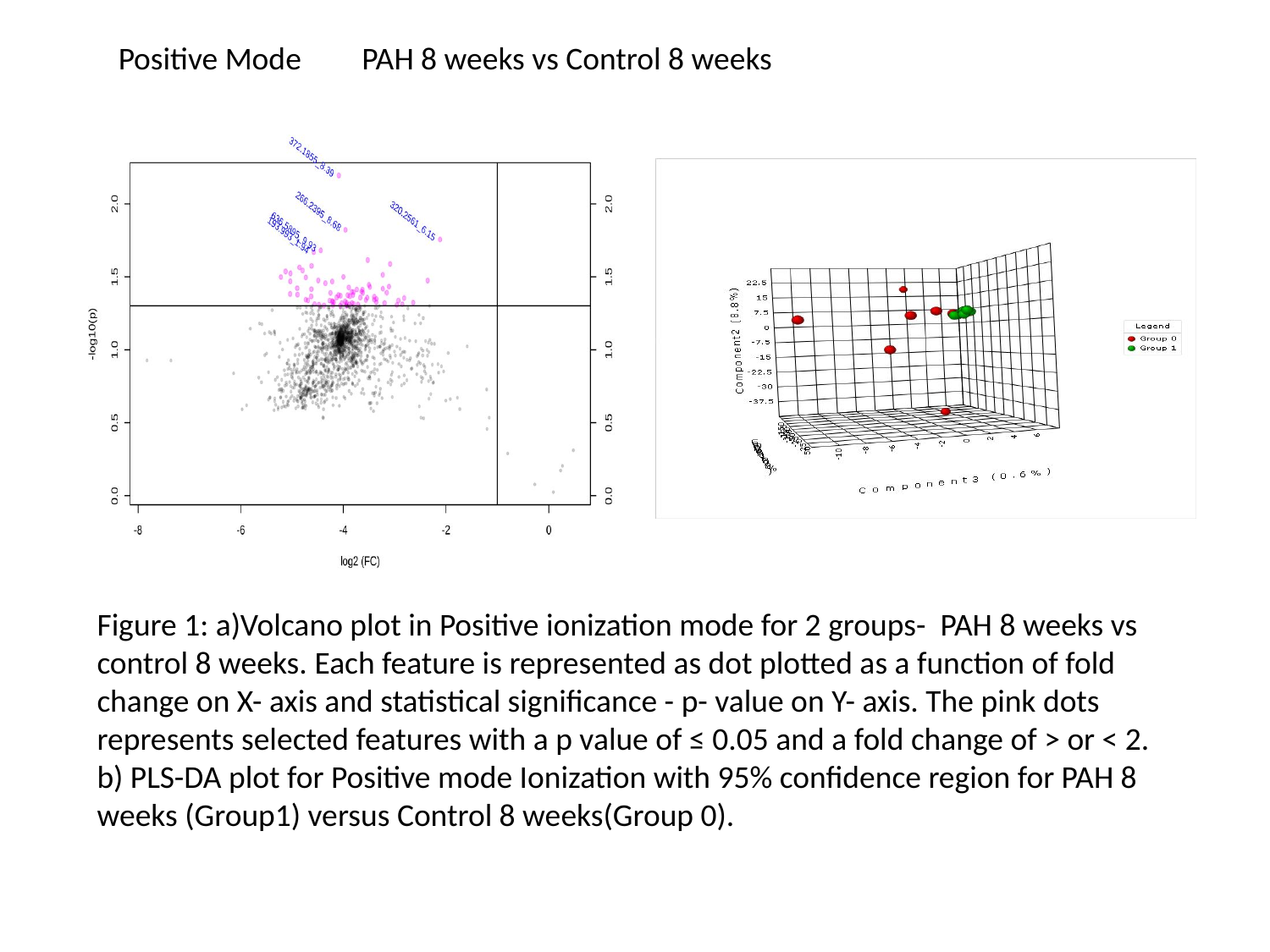

Positive Mode
PAH 8 weeks vs Control 8 weeks
Figure 1: a)Volcano plot in Positive ionization mode for 2 groups- PAH 8 weeks vs control 8 weeks. Each feature is represented as dot plotted as a function of fold change on X- axis and statistical significance - p- value on Y- axis. The pink dots represents selected features with a p value of ≤ 0.05 and a fold change of > or < 2. b) PLS-DA plot for Positive mode Ionization with 95% confidence region for PAH 8 weeks (Group1) versus Control 8 weeks(Group 0).

## Slide 9
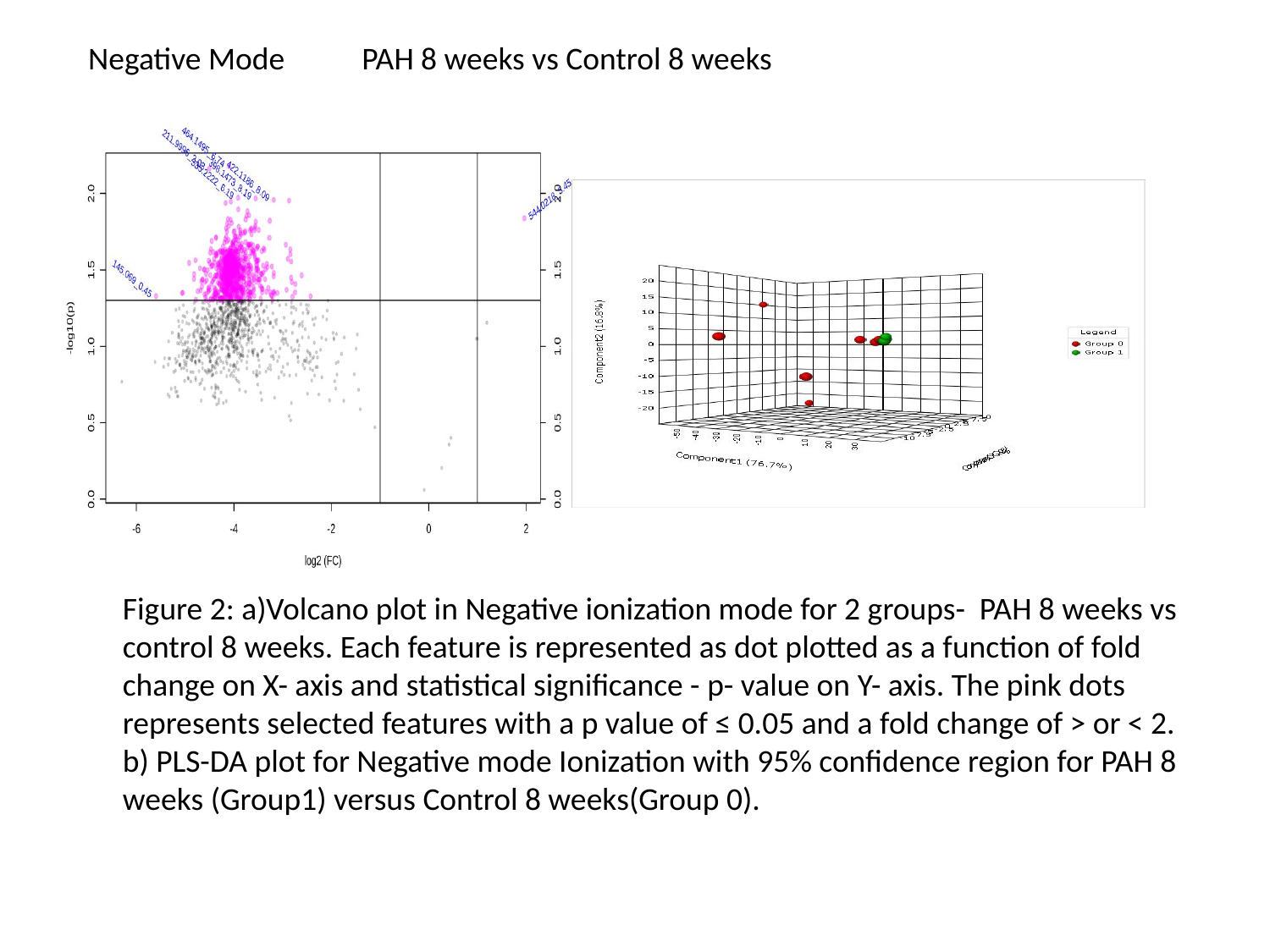

Negative Mode
PAH 8 weeks vs Control 8 weeks
Figure 2: a)Volcano plot in Negative ionization mode for 2 groups- PAH 8 weeks vs control 8 weeks. Each feature is represented as dot plotted as a function of fold change on X- axis and statistical significance - p- value on Y- axis. The pink dots represents selected features with a p value of ≤ 0.05 and a fold change of > or < 2. b) PLS-DA plot for Negative mode Ionization with 95% confidence region for PAH 8 weeks (Group1) versus Control 8 weeks(Group 0).

## Slide 10
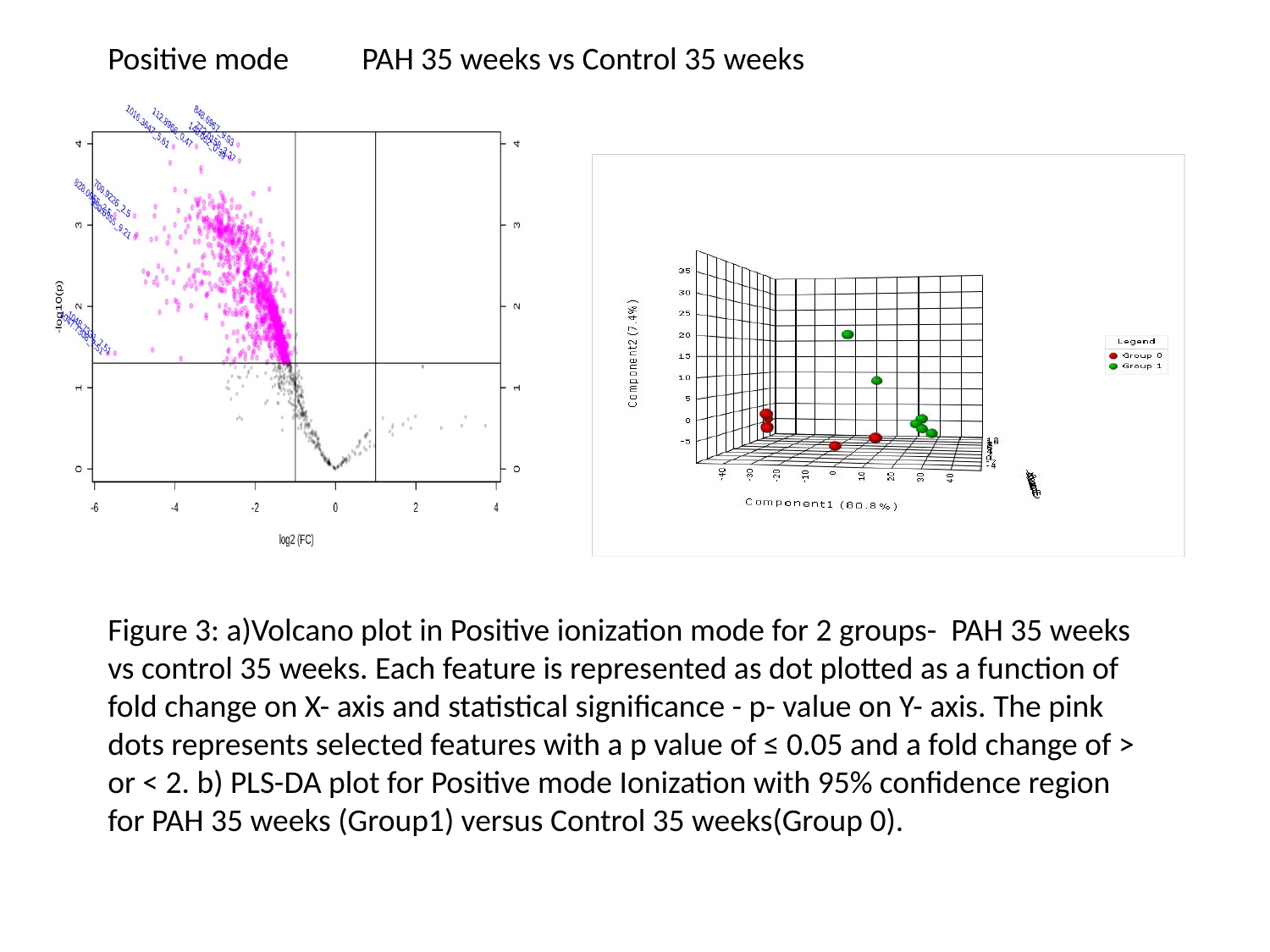

Positive mode
PAH 35 weeks vs Control 35 weeks
Figure 3: a)Volcano plot in Positive ionization mode for 2 groups- PAH 35 weeks vs control 35 weeks. Each feature is represented as dot plotted as a function of fold change on X- axis and statistical significance - p- value on Y- axis. The pink dots represents selected features with a p value of ≤ 0.05 and a fold change of > or < 2. b) PLS-DA plot for Positive mode Ionization with 95% confidence region for PAH 35 weeks (Group1) versus Control 35 weeks(Group 0).

## Slide 11
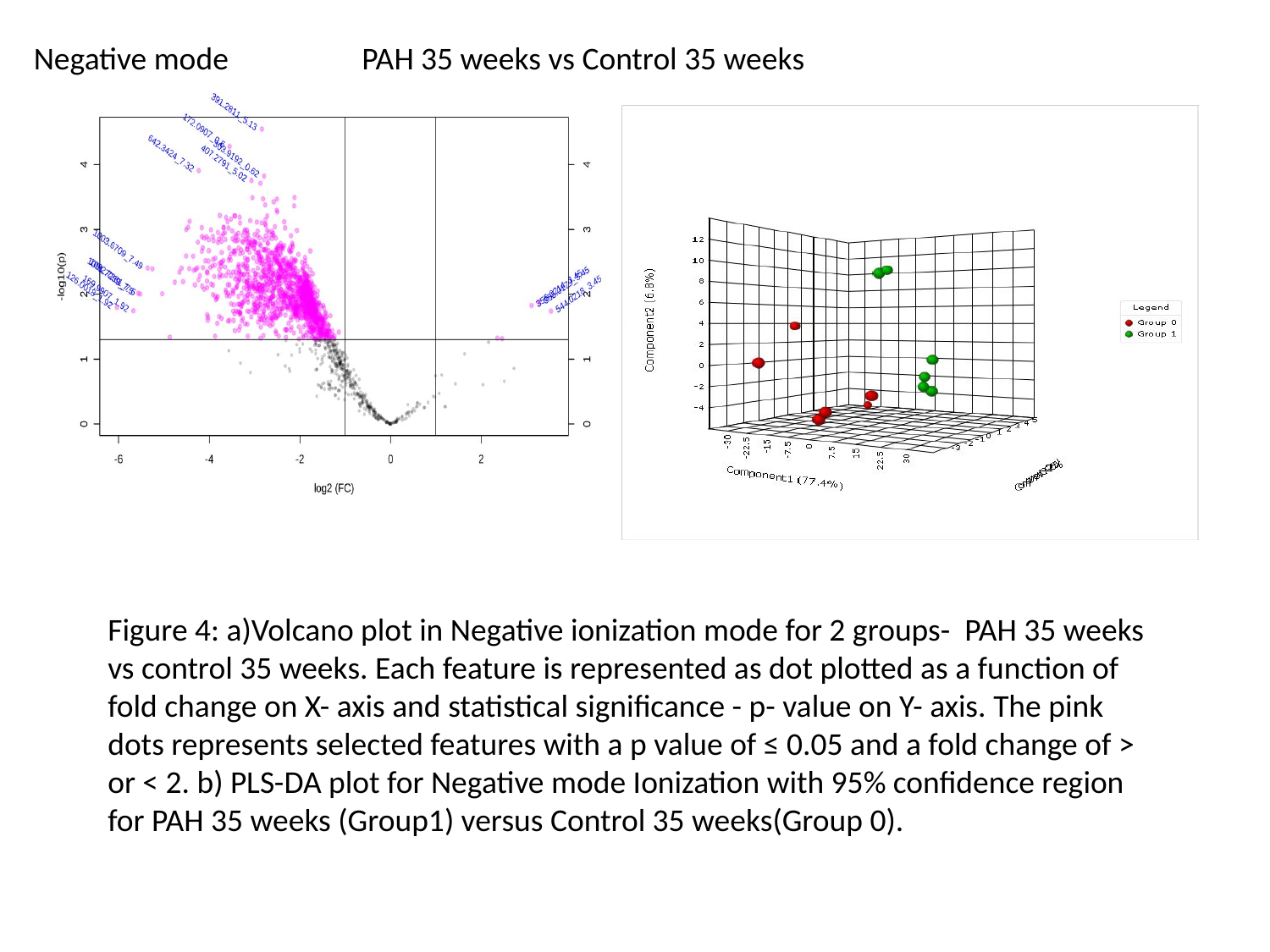

Negative mode
PAH 35 weeks vs Control 35 weeks
Figure 4: a)Volcano plot in Negative ionization mode for 2 groups- PAH 35 weeks vs control 35 weeks. Each feature is represented as dot plotted as a function of fold change on X- axis and statistical significance - p- value on Y- axis. The pink dots represents selected features with a p value of ≤ 0.05 and a fold change of > or < 2. b) PLS-DA plot for Negative mode Ionization with 95% confidence region for PAH 35 weeks (Group1) versus Control 35 weeks(Group 0).

## Slide 12
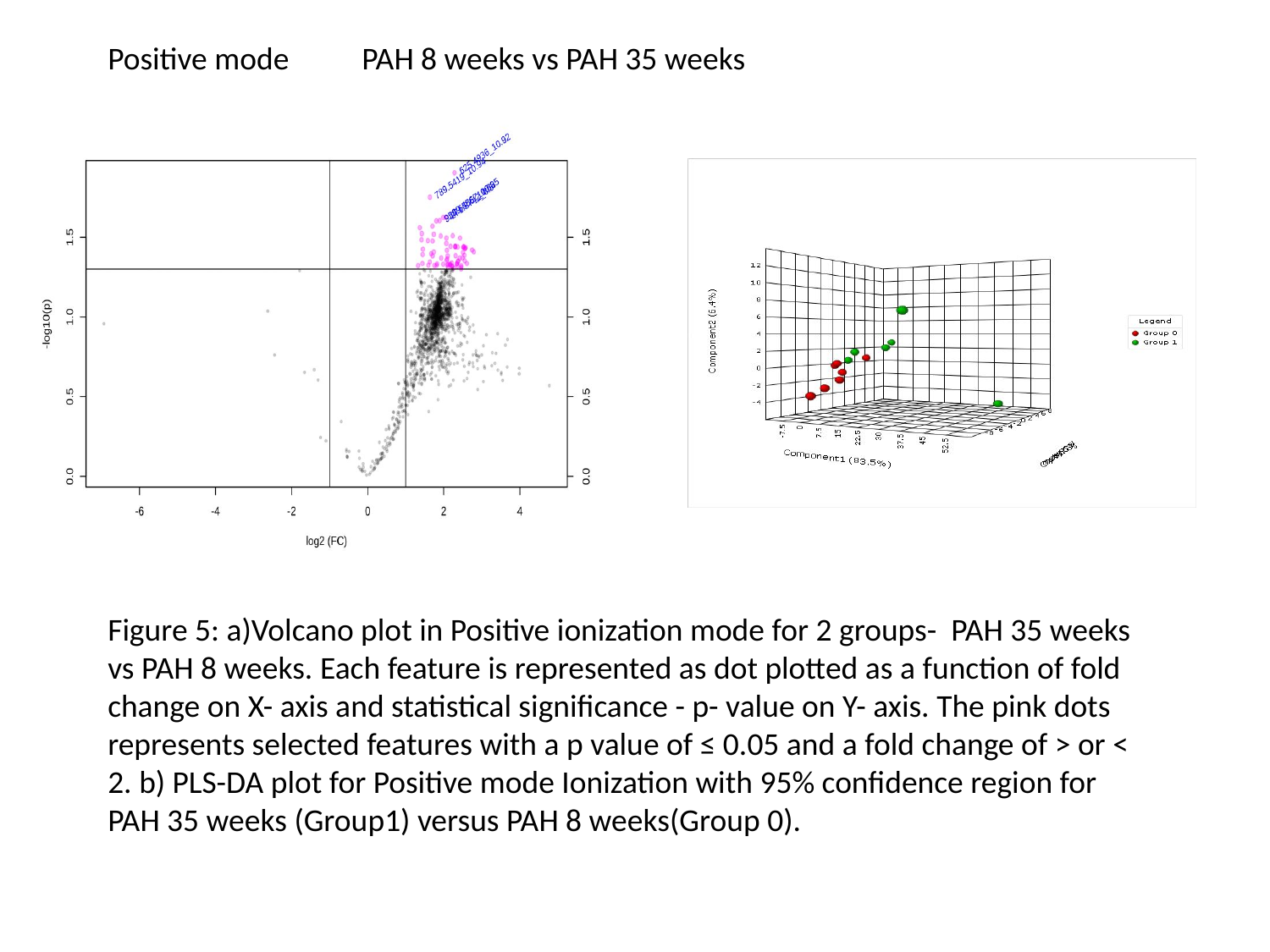

Positive mode
PAH 8 weeks vs PAH 35 weeks
Figure 5: a)Volcano plot in Positive ionization mode for 2 groups- PAH 35 weeks vs PAH 8 weeks. Each feature is represented as dot plotted as a function of fold change on X- axis and statistical significance - p- value on Y- axis. The pink dots represents selected features with a p value of ≤ 0.05 and a fold change of > or < 2. b) PLS-DA plot for Positive mode Ionization with 95% confidence region for PAH 35 weeks (Group1) versus PAH 8 weeks(Group 0).

## Slide 13
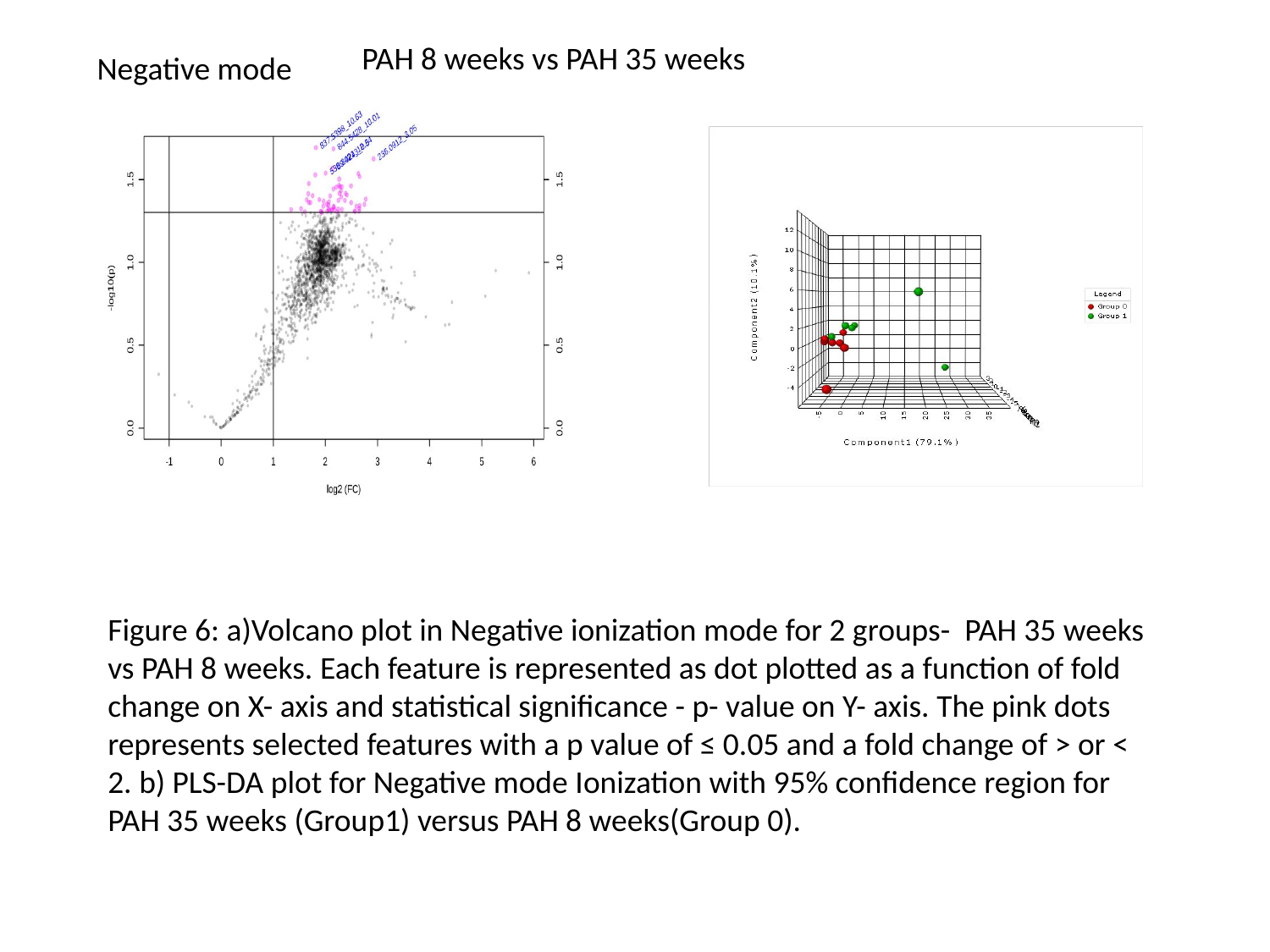

PAH 8 weeks vs PAH 35 weeks
Negative mode
Figure 6: a)Volcano plot in Negative ionization mode for 2 groups- PAH 35 weeks vs PAH 8 weeks. Each feature is represented as dot plotted as a function of fold change on X- axis and statistical significance - p- value on Y- axis. The pink dots represents selected features with a p value of ≤ 0.05 and a fold change of > or < 2. b) PLS-DA plot for Negative mode Ionization with 95% confidence region for PAH 35 weeks (Group1) versus PAH 8 weeks(Group 0).

## Slide 14
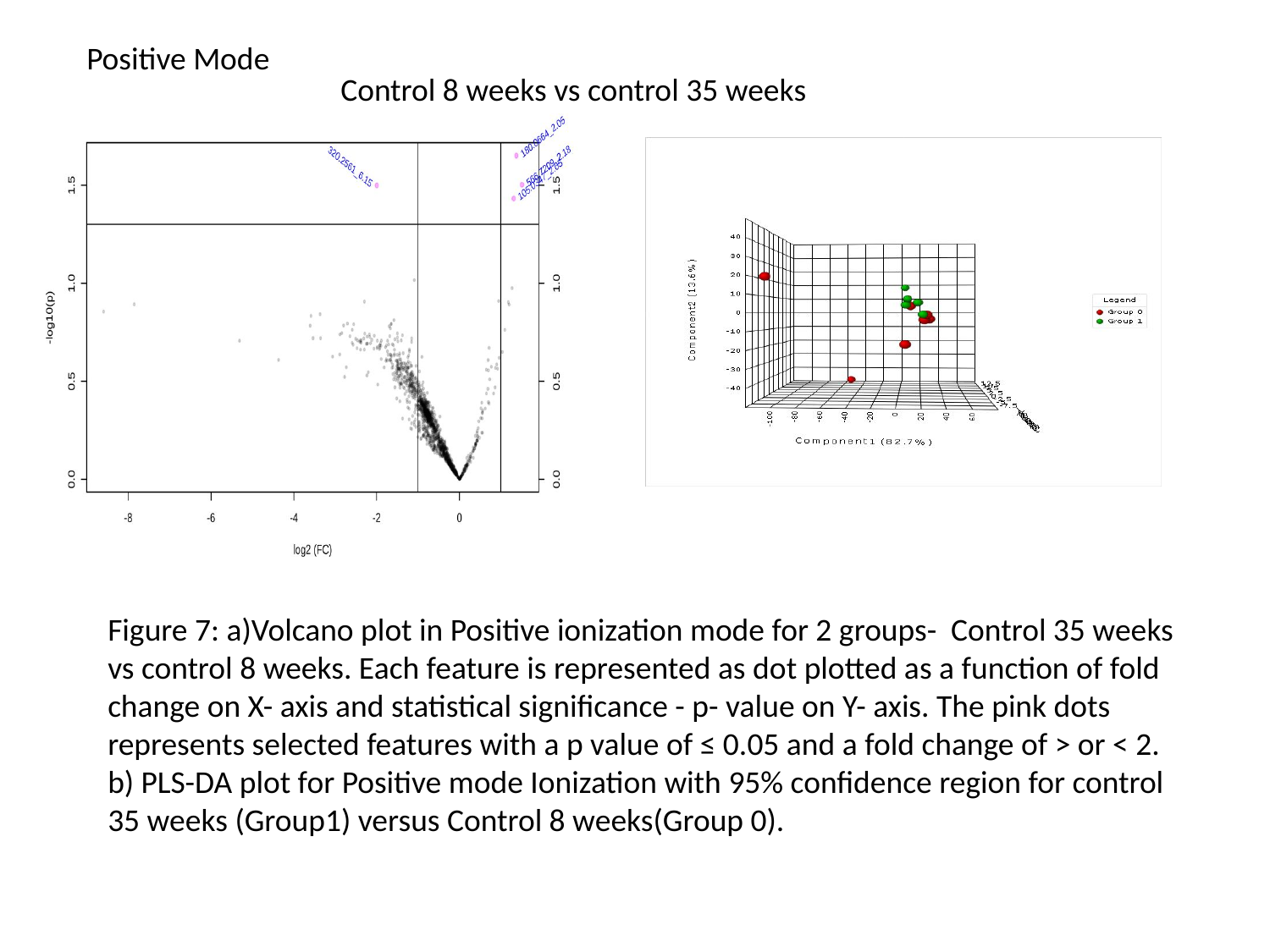

Positive Mode
Control 8 weeks vs control 35 weeks
Figure 7: a)Volcano plot in Positive ionization mode for 2 groups- Control 35 weeks vs control 8 weeks. Each feature is represented as dot plotted as a function of fold change on X- axis and statistical significance - p- value on Y- axis. The pink dots represents selected features with a p value of ≤ 0.05 and a fold change of > or < 2. b) PLS-DA plot for Positive mode Ionization with 95% confidence region for control 35 weeks (Group1) versus Control 8 weeks(Group 0).

## Slide 15
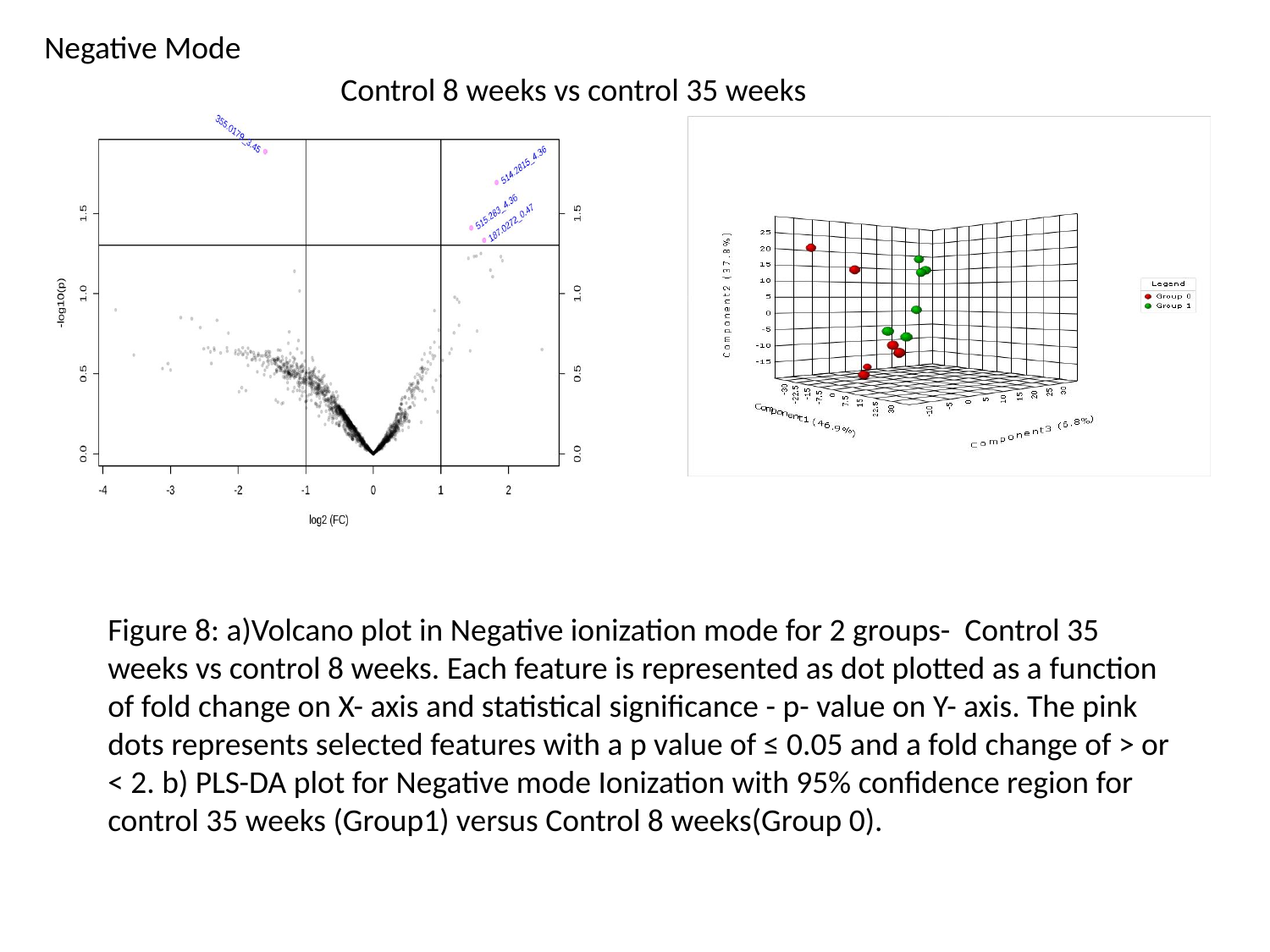

Negative Mode
Control 8 weeks vs control 35 weeks
Figure 8: a)Volcano plot in Negative ionization mode for 2 groups- Control 35 weeks vs control 8 weeks. Each feature is represented as dot plotted as a function of fold change on X- axis and statistical significance - p- value on Y- axis. The pink dots represents selected features with a p value of ≤ 0.05 and a fold change of > or < 2. b) PLS-DA plot for Negative mode Ionization with 95% confidence region for control 35 weeks (Group1) versus Control 8 weeks(Group 0).
